# S100A10 Has a Critical Regulatory Function in Mammary Tumor Growth and Metastasis: Insights Using MMTV-PyMT Oncomice and Clinical Patient Sample Analysis

**DOI:** 10.3390/cancers12123673

**Published:** 2020-12-07

**Authors:** Alamelu G. Bharadwaj, Margaret L. Dahn, Rong-Zong Liu, Patricia Colp, Lynn N. Thomas, Ryan W. Holloway, Paola A. Marignani, Catherine K. L. Too, Penelope J. Barnes, Roseline Godbout, Paola Marcato, David M. Waisman

**Affiliations:** 1 Department of Pathology, Dalhousie University, Halifax, NS B3H 4R2, Canada; Alamelu.Bharadwaj@dal.ca (A.G.B.); meg.thomas@dal.ca (M.L.D.); p.colp@dal.ca (P.C.); penny.barnesmd@nshealth.ca (P.J.B.); paola.marcato@dal.ca (P.M.); 2 Department of Oncology, University of Alberta, Edmonton, AB T6G 2Z1, Canada; rongzong@ualberta.ca (R.-Z.L.); rgodbout@ualberta.ca (R.G.); 3 Department of Biochemistry & Molecular Biology, Dalhousie University, Halifax, NS B3H 4R2, Canada; Lynn.Thomas@Dal.Ca (L.N.T.); Ryan.W.Holloway@Dal.Ca (R.W.H.); paola.marignani@dal.ca (P.A.M.); catherine.too@dal.ca (C.K.L.T.); 4 Department of Microbiology and Immunology, Dalhousie University, NS B3H 4R2, Canada

**Keywords:** breast cancer, S100A10 (p11), tumor growth, tumor progression, macrophages, metastasis, carcinoma, mammary gland, triple negative

## Abstract

**Simple Summary:**

The key challenges that face patients during breast cancer therapy is the metastatic spread and aggressiveness of the disease. Thus, the goal of current breast cancer research is to discover new therapeutic and diagnostic targets that limit the aggressive spread of the cancer. In this study, we investigated the role of protein S100A10 (p11) in breast tumor growth, progression, and metastasis using mouse cancer models and patient tumor sample analysis. We have demonstrated in our previous studies that p11 is critical for the function of a proteolytic enzyme–plasmin, which aids in the digestion of the tissues surrounding the tumor and allows the escape of the cancer cells from the breast tissue to organs such as the lungs and bone. Here, we present evidence that genetic deletion of p11 results in smaller and less aggressive mammary tumors in mice. We also observed that the cancer spread to the lungs is dramatically reduced in the absence of p11 gene in mice. Subsequent analysis of breast cancer patient tissues showed a correlation between higher p11 expression and both poor survival and aggressive cancer.

**Abstract:**

S100A10 (p11) is a plasminogen receptor that regulates cellular plasmin generation by cancer cells. In the current study, we used the MMTV-PyMT mouse breast cancer model, patient tumor microarray, and immunohistochemical (IHC) analysis to investigate the role of p11 in oncogenesis. The genetic deletion of p11 resulted in significantly decreased tumor onset, growth rate, and spontaneous pulmonary metastatic burden in the PyMT/p11-KO (knock-out) mice. This phenotype was accompanied by substantial reduction in Ki67 positivity, macrophage infiltration, decreased vascular density in the primary tumors, and decrease in invasive carcinoma and pulmonary metastasis. Surprisingly, IHC analysis of wild-type MMTV-PyMT mice failed to detect p11 expression in the tumors or metastatic tumor cells and loss of p11 did not decrease plasmin generation in the PyMT tumors and cells. Furthermore, tumor cells expressing p11 displayed dramatically reduced lung metastasis when injected into p11-depleted mice, further strengthening the stromal role of p11 in tumor growth and metastasis. Transcriptome analysis of the PyMT tumors from p11-KO mice showed marked reduction in genes such as *Areg*, *Muc1*, and *S100a8* involved in breast cancer development, progression, and inflammation. The PyMT/p11-KO tumors displayed a remarkable increase in inflammatory cytokines such as interleukin (*Il*)-*6*, *Il-10*, and interferon (*Ifn*)-*γ*. Gene expression profiling and IHC of primary breast cancer samples showed that p11 mRNA and protein levels were significantly higher in tumor tissues compared to normal mammary tissue. P11 mRNA expression was significantly associated with poor patient prognosis and significantly elevated in high grade, triple negative (TN) tumors, and tumors with high proliferative index. This is the first study examining the crucial role of p11 in breast tumor development and metastasis, thus emphasizing its potential as a diagnostic and prognostic biomarker in breast cancer.

## 1. Introduction

Breast cancer is the leading cause of cancer death among women worldwide. The vast number of cancer-associated deaths are due to metastases rather than primary disease alone. Currently, strategies to treat and eliminate metastasis are limited and challenged by resistance to therapies. Thus, discovering the underlying molecular mechanisms that promote breast cancer metastasis and therapy resistance is critical for identifying novel treatment strategies.

Extracellular proteases promote degradation of the extracellular matrix and form a key component of the cascade of events contributing to cancer cell invasion and metastasis. [1,2,3]. Increasing evidence has shown elevated expression of proteases plasmin and matrix metalloproteases (MMPs) in breast cancer progression and has been extensively studied with mouse tumor models [4]. Plasminogen (Plg), synthesized by the liver is activated to plasmin (Pm) by tissue plasminogen activator (tPA) and urokinase plasminogen (uPA) activator, (reviewed in [5,6]). This process, normally slow, is accelerated by plasminogen receptors (PgR) on the cell surface. Our laboratory identified S100A10 (p11) as a plasminogen receptor that forms a complex with both plasminogen and plasminogen activator [7,8,9,10].

P11 is a multifunctional protein and a member of the S100 proteins. Several extracellular and intracellular functions have been identified for p11; the plasminogen receptor function is the most well studied so far [5]. P11, present on the extracellular surface, is complexed with its binding partner annexin A2 (p36). We have reported that p11 regulates plasminogen activation on the cell surface of many cancer cells including fibrosarcoma, colorectal, lung, and pancreatic cells [8,11,12,13,14,15,16]. We have also reported that p11 regulates the plasmin production of stromal cells, including macrophages and endothelial cells [17,18,19], and that p11-dependent plasmin generation is necessary for macrophage infiltration to the site of inflammation in subcutaneous tumor growth.

Several studies have shown correlations between p11 gene expression and poor prognosis and overall survival in lung [20,21,22], colorectal, ovarian, kidney, and gastric cancers, anaplastic thyroid carcinoma, melanoma and acute lymphoblastic leukemia (reviewed in [12]), and pancreatic ductal adenocarcinoma [16]. P11 is upregulated in basal-type breast cancer [23] and during the process of intravasation and epithelial mesenchymal transition [24].

Although we have shown that p11 is important for ectopic tumor growth in both NOD/SCID (Non-obese diabtetic/severe combined immunodeficiency ) and syngeneic mouse models for various cancer cell lines such as colorectal, fibrosarcoma, and pancreatic cancer, these studies have limitations. Firstly, they do not reproduce the complex multistep landscape of human oncogenesis. Secondly, the growth, invasion, and metastasis of cancer also depends on its interaction with the tumor microenvironment making it difficult to distinguish the individual contribution of tumor and stromal cells to cancer progression. To circumvent these experimental challenges, and to advance our understanding of the role of p11 in oncogenesis, we have established the MMTV-PyMT (mouse mammary tumor virus-polyoma middle tumor-antigen) transgenic breast cancer model in wild-type and p11 knockout mice and have used this double transgenic model to investigate the role of p11 in breast cancer malignancy.

In the MMTV-PyMT transgenic mouse model, mammary gland specific expression of the oncogene PyMT under the MMTV promoter, results in widespread tumor growth in all ten mammary glands and spontaneous metastasis to the lymph nodes and lungs. This occurs with a mean latency of 92 days, with high penetrance and almost a 100% incidence of metastasis. This mouse model is very similar to human breast cancer in that the tumors display histological and molecular characteristics mirroring the progression of human breast cancer [25,26,27] and have a reactive stroma. This model has been widely used to establish the role of proteolytic activity in cancer cell malignancy. Proteases that affect metastasis in this model system include Plg [28,29], uPA [30], MMP-9 and MMP-3 [4,31,32], cathepsin B [33,34], and ADAMTS1 (short for disintegrin and metalloproteinase with thrombospondin motifs) [35]. Another important feature of the MMTV-PyMT model is that the increased metastatic potential is largely dependent on the presence of macrophages in the primary tumor [36,37]. Thus, the MMTV-PyMT model is a pertinent model to investigate the functional role of p11.

In the current study, we conducted a systematic, functional, and correlative analysis of the role of p11 in breast cancer oncogenesis using the MMTV-PyMT transgenic mouse model. We have also performed gene expression profiling and protein expression analysis of human breast cancer tissues. The studies described herein indicate that p11 plays a complex and multifunctional role in breast tumor growth, progression, and metastasis.

## 2. Results

### 2.1. Loss of p11 Results in Delayed Appearance of Multifocal Dysplastic Lesions

**Figure 1:**
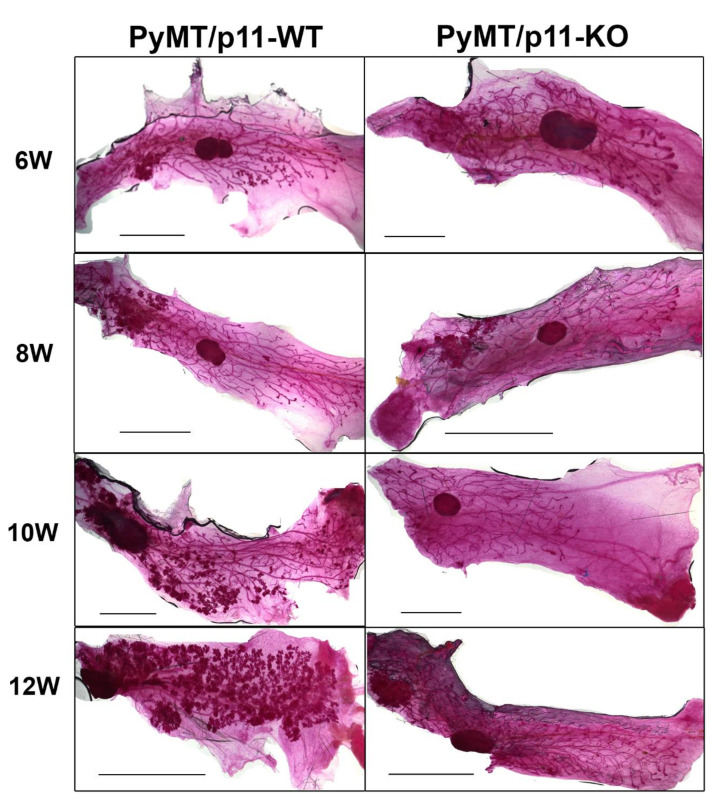
Loss of p11 affects formation of hyperplastic lesions in PyMT driven tumors. Whole mammary glands from PyMT/p11-WT (n = 3) and PyMT/p11-KO mice at (6 (6 W), 8 (8 W), 10 (10 W), 12 (12 W) weeks) were excised, fixed, and stained with carmine alum as per standard protocols (See Materials and Methods). Representative images from the 4th abdominal mammary gland from each group and time point are shown. Scale bar 2 mm.

The fourth inguinal mammary gland from PyMT/p11-WT (wild-type) and PyMT/p11-KO (knock-out) mice was isolated at 8, 10, and 12 weeks of age, fixed and stained with carmine alum [37]. As early as 6 weeks of age, we observed the appearance of early hyperplastic lesion at a single focus or in some cases, multiple foci beneath the nipple in the older zone of the ductal tree in the PyMT/p11-WT.. At 8 weeks, we observed the formation of multiple small nodules in the distal newer ducts, which then spread extensively to the entire length of mammary gland by 12 weeks of age. In contrast, PyMT/p11-KO mice showed complete absence of hyperplastic tumor foci at 6 weeks, which appeared first at 8 weeks of age, and were restricted to only a fraction of the older ductal structures even at 12 weeks. The most dramatic difference was the complete absence of multiple tumor foci throughout the mammary gland of the PyMT/p11-KO mice at 12 weeks (Figure 1). Hematoxylin and Eosin (H&E) staining of a representative tissue section (5 µm) (Figure S1) showed increased hyperplastic lesions in the PyMT/p11-WT as age progressed from 6 weeks to 12 weeks, whereas none were observed in the PyMT/p11-KO tumors.

### 2.2. p11 Plays a Role in Mammary Tumor Growth and Progression

We employed a cohort of 27 PyMT/p11-WT and 28 PyMT/p11-KO mice and monitored the time of appearance and size of palpable tumors. A fraction of the mice was sacrificed at 20 weeks and the total tumor burden was determined. The remaining mice were monitored further until a combined tumor volume of 4000 mm^3^ was attained, the humane endpoint of the experiments.

**Figure 2:**
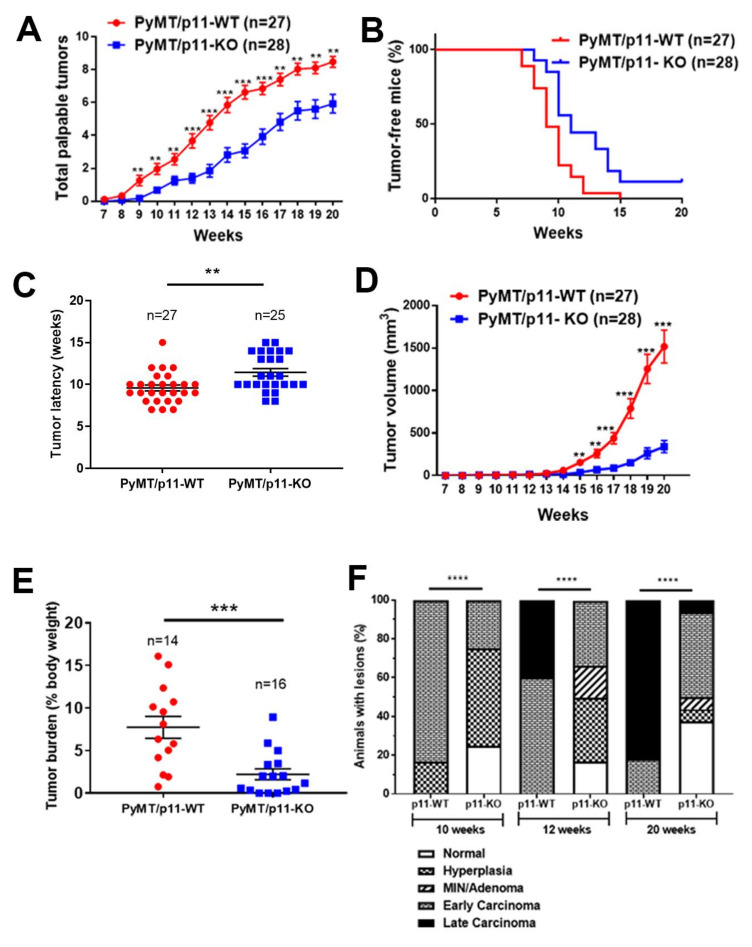
Tumor initiation, growth, burden, and progression are decreased and delayed in PyMT/p11-KO mice. (**A**) Appearance of palpable tumors was monitored weekly. (**B**) Percent tumor-free mice was calculated using Kaplan–Meir analysis (hazard ratio (HR)—2.24, *p* = 0.0004). (**C**) Tumor latency was plotted for each mouse based on the first appearance of palpable tumor. (**D**) Tumor volume was measured weekly using calipers. Total volume was plotted to represent tumor growth rate. (**E**) Total tumor weight at endpoint (20 weeks) was determined as a percentage of body weight. Data are a compilation of three independent experiments, except (**D**), which shows data from one independent experiment. Significance was determined by non-parametric *t*-test (Mann Whitney U test–unpaired, non-parametric test for means). (**F**) Mammary glands and tumors from PyMT/p11-WT and PyMT/p11-KO mice were harvested at 20 weeks, formalin-fixed, embedded, and sectioned at 5 µm thickness. The H&E and smooth muscle α-actin-stained sections were classified into histopathological stages by a pathologist in a blinded manner. (n = 6 to 12 mice in each group). Statistical analysis was performed by χ^2^-test (*p* < 0.0001). The ssterix on each plot represents *p*-values as follows. ** *p* < 0.01, *** *p* < 0.001, **** *p* < 0.0001.

The first palpable tumor appeared at 9 weeks in the PyMT/p11-WT mice compared to 11 weeks in the PyMT/p11-KO mice (Figure 2A). At 10 weeks, 77% of PyMT/p11-WT and 42% of PyMT/p11-KO mice developed palpable tumors. All the PyMT/p11-WT mice developed palpable tumors by 105 days of age (15 weeks), whereas only 89% of PyMT/p11-KO mice developed tumors at 20 weeks (Figure 2B). Overall, the mean tumor latency was increased by two weeks in the PyMT/p11-KO mice (Figure 2C). The tumor volume was dramatically decreased in the PyMT/p11-KO mice with a 4- and 6-fold decrease at 15 and 20 weeks, respectively (Figure 2D).

Histopathological progression to late carcinoma stage was delayed in PyMT/p11-KO mice (Figure 2E). By 10 weeks of age, 83% of the PyMT/p11-WT mice and only 25% of the PyMT/p11-KO progressed to the early carcinoma stage. By 20 weeks, 82% of the PyMT/p11-WT mice and 6.25% of the PyMT/p11-KO had progressed to the late carcinoma stage. Interestingly, at this endpoint, 37.5% of PyMT/p11-KO mice showed normal mammary gland histopathology.

### 2.3. Loss of p11 Reduces Tumor Cell Proliferation, Vascular Density, and Macrophage Infiltration

**Figure 3:**
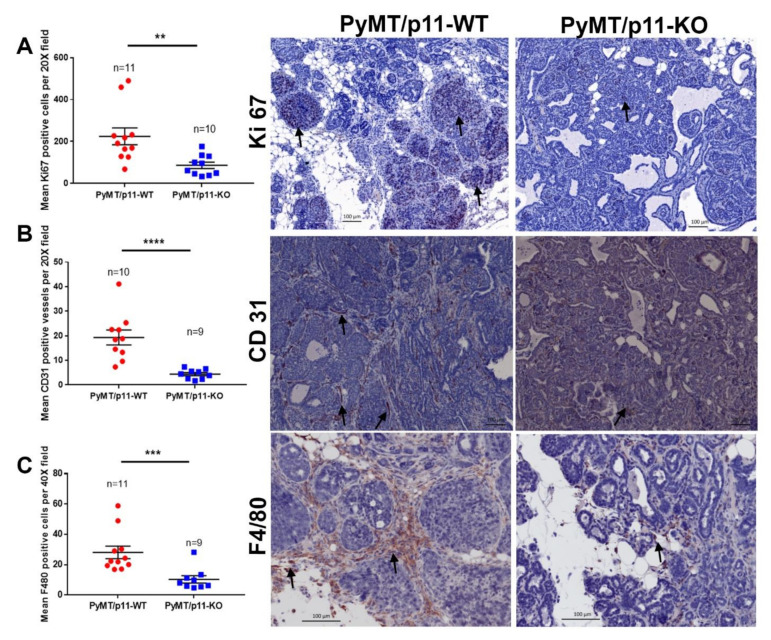
PyMT/p11-KO mice tumors show reduced proliferation, vascular density and macrophage infiltration. Formalin-fixed, paraffin-embedded, and sectioned tissues from PyMT/p11-WT (n = 11) and PyMT/p11-KO (n = 10) (end-point, 20 weeks) mice were immunostained for (**A**) Ki67 (proliferation marker), (**B**) CD31 (endothelial marker), and (**C**) F4/80 (mouse macrophage marker). (**A**) Immunostaining using anti-rabbit Ki67 antibody (Abcam, Cambridge, MA, USA). Stained sections were imaged using Zeiss Axio Imager Z1 W/color and monochrome camera (Carl Zeiss Canada, Toronto, ON, Canada) at 10× magnification. Left panel: The number of Ki67-positive cells was manually counted using Zen (2012) software in 7–10 random fields per tissue section/mouse. Significance was determined using Mann Whitney U test (unpaired, non-parametric test for means), *p* = 0.0021. Right panel: The representative image was captured at 20× magnification. Scale bar–100 µm. (**B**) Immunostaining using anti-rabbit CD31 antibody (Abcam, Cambridge, MA, USA). Stained sections were imaged using Aperio Scanning system (Leica Biosystems, Concord, Ontario) at 40× magnification. Left panel: The number of CD31-positive cells were manually counted using Imagescope software (Leica Biosystems, Concord, Ontario) in 7–10 random fields per tissue section/mouse. Mann Whitney U test (unpaired, non-parametric test for means) shows *p* value < 0.0001. Right panel: Representative image at 20× magnification. Scale bar–100 µm. (**C**) Immunostaining using anti-rat F4/80 antibody (BM8, Thermofisher Scientific, Waltham, MA, USA). Stained sections were imaged as in (**A**), but at 40× magnification. Left panel: The number of F4/80-positive cells were manually counted as in (**A**). Mann Whitney U test (unpaired, non-parametric test for means) showed *p* < 0.0001. Right panel: Representative image at 20× magnification. Scale bar–100 µm. The asterix on each plot represents *p*-values as follows. ** *p* < 0.01, *** *p* < 0.001, **** *p* < 0.0001.

The proliferative index of PyMT/p11-WT and PyMT/p11-KO tumors at endpoint (20 weeks) were measured using Ki67 positive immunoreactivity [38]. We observed a 2.9-fold reduction in mean Ki67 positive-cells in tumors from PyMT/p11-KO mice, suggesting that p11 functions in modulation of tumor cell proliferation (Figure 3A). Although there was a trend for increased apoptosis in the PyMT/p11-KO tumors, this was not of statistical significance (Figure S4A). We observed a 6-fold reduction in endothelial cell staining (CD31-positive) in the PyMT/p11-KO tumors compared to PyMT/p11-WT tumors (Figure 3B). In both groups of PyMT mice, the macrophages (F4/80-positive) were restricted to the peripheral area of the tumors. However, there was a significant (11.5-fold) decrease in the macrophage density in the tumors from PyMT/p11-KO mice compared to the PyMT/p11-WT mice (Figure 3C). We also observed a qualitative increase in CD3+ (total T cells) staining in the tumors, but this difference was not statistically significant (Figure S4B).

### 2.4. Loss of p11 Reduced Spontaneous and Experimental Metastasis

**Figure 4:**
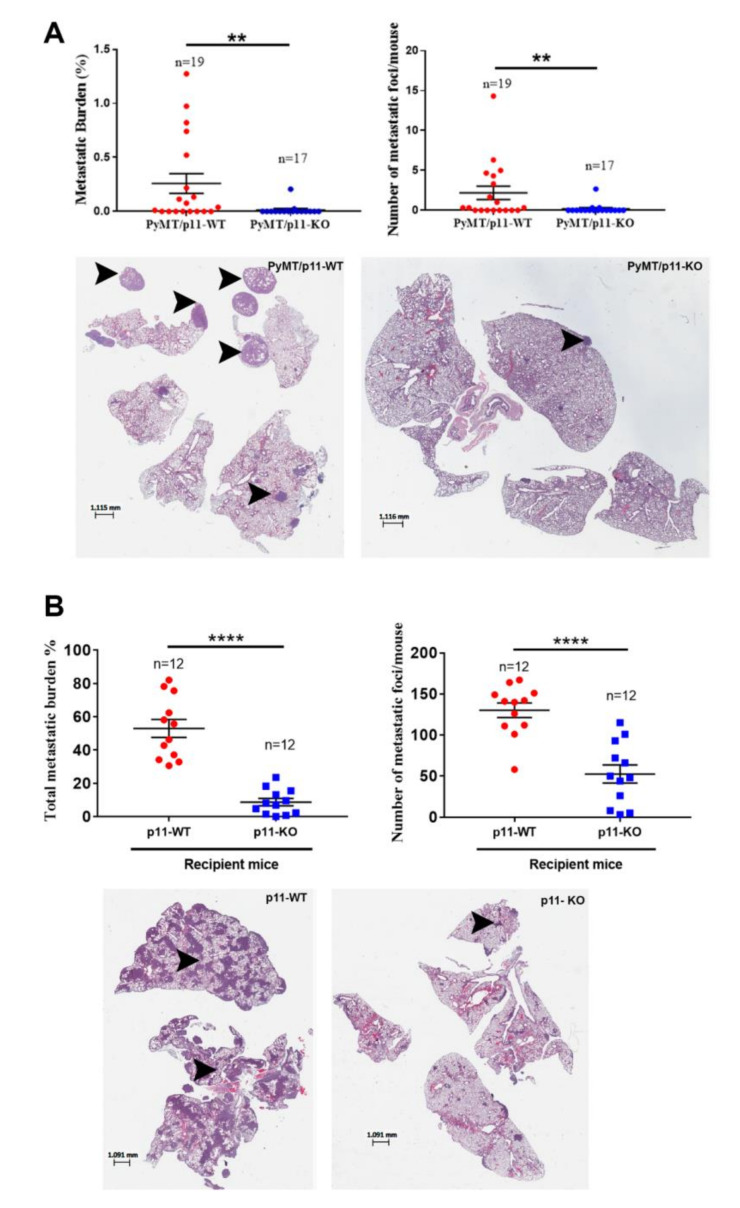
Metastasis is diminished in PyMT/p11-KO mice. (**A**) We evaluated pulmonary metastasis at the 20-week end point in (spontaneous model) PyMT/p11-WT and PyMT/p11-KO mice by microscopic examination of formalin-fixed, H&E-stained lung sections (5 µm). Three lung sections each 100 µm apart were used for staining. Quantification was performed using Aperio image analysis software (Imagescope). Mean values for three sections were used to calculate the metastatic burden and foci values. (**A**) We evaluated the metastatic burden by measuring the total metastatic area and total lung area, followed by normalization (A—upper left panel). Mann Whitney U test showed statistical significance with *p* value of 0.0062. The number of metastatic foci per mouse lung section was determined by manual counting of images (A—upper right panel). Mann Whitney U test showed *p* value of 0.0069. Lower panels: Representative lung images from WT and KO mice. (**B**) Experimental metastasis assay. We injected 2.5 × 10^5^ Py8119 (p11-WT levels) cells into p11-WT and p11-KO mice (n = 12 mice per group). The lungs were harvested after 14 days, formalin-fixed, and sectioned at 5 µm as described above. Mann Whitney non-parametric *t*-test (for means) for statistical significance was performed. Metastasis was pooled and combined from two independent experiments (n = 6 each). *p* < 0.0001. Lower panels: representative lung images from WT and KO mice. The asterix on each plot represents p-values as follows. ** *p* < 0.01, **** *p* < 0.0001.

We harvested the lungs from 20-week old mice and performed histochemical analysis. We observed a significant 18-fold decrease in metastatic burden and a 14-fold decrease in number of metastatic foci (Figure 4A) in the PyMT/p11-KO mice. Furthermore, the presence of metastatic foci was observed only in 3 of 17 (17.6%) in the PyMT/p11-KO mice compared to 10 of 19 (53%) in the PyMT/p11-WT mice, suggesting an important role for p11 in spontaneous metastasis. Next, we injected the PyMT transformed cell line, Py8119 with WT p11 levels intravenously (lateral tail vein) in p11-WT and p11-KO mice. We observed a dramatic decrease in the metastatic burden (6-fold) and number of metastatic foci (2.5-fold) in the p11-KO mice (Figure 4B). These results suggest that stromal p11 is important for the extravasation process and establishment of metastases by these breast cancer cells.

### 2.5. p11 Expression Is Restricted to the Stromal Compartment in Mammary and Pulmonary Metastatic Tumors

**Figure 5:**
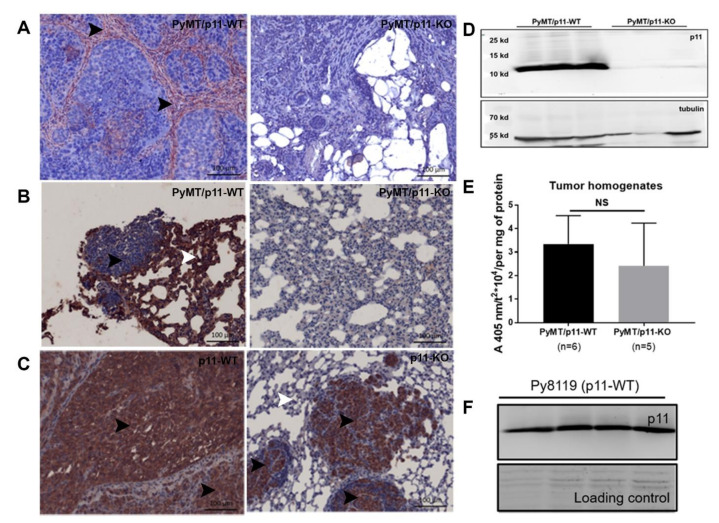
Expression of p11 in mammary and pulmonary metastatic tumors is restricted to the stromal compartment in the PyMT/p11-WT tumors. IHC staining was performed using anti-rabbit p11 antibody (Proteintech, Rosemont, IL, USA), on 5 µm sections from 20-week end-point PyMT/p11-WT (n = 10) and PyMT/p11-KO tumors (n = 3), and lungs from spontaneous and experimental metastasis assay. (**A**) Representative images of p11 immunostained sections of PyMT mammary tumors. As anticipated, the tumors from PyMT/p11-KO mice showed no staining, validating the specificity of the antibody. (**B**) PyMT spontaneous lung metastasis from PyMT/p11-WT and PyMT/p11-KO mice, and (**C**) experimental metastasis of Py8119 cells injected in p11-WT (n = 4) and p11-KO (n = 4) mice are shown. Scale bar is 100 µm. (**D**) Western blot of p11 expression in total cell tumor homogenates from PyMT/p11-WT (n = 3) and PyMT/p11-KO mice (n = 3). (**E**) Fresh and frozen tumors from both PyMT/p11-WT (n = 6 mice) and PyMT/p11-KO (n = 5 mice) were homogenized and equal protein (30–60 µg) was used for plasmin generation assay as described in Materials and Methods and Supplemental Section. Mann Whitney U (unpaired, non-parametric test for means) *t*-test show *p* =0.3290). NS, not significant (**F**) Western blot for p11 expression in total cell lysates in Py8119 cells obtained from ATCC (American Type Culture Collection) (n = 4) using goat anti-mouse antibody (R&D systems, Minneapolis, MN, USA).

To further investigate the expression and localization of p11 in the PyMT tumor and stromal compartment, we performed immunohistochemical analysis of tumors from both PyMT/p11-WT and PyMT/p11-KO cohorts. Interestingly, we observed that the majority of the p11 staining was localized to the stromal compartment in the PyMT/p11-WT mice. Only a fraction (33%) of tumors examined presented with diffuse p11 staining in the tumor cells (Figure 5A).

To further evaluate if p11 expression was induced in the metastatic tumor cells, we immunostained lung sections from PyMT/p11-WT mice and observed that the lung tissue but not the metastatic foci showed immunoreactivity for p11. (Figure 5B). This suggested that the tumor cells in the PyMT tumors do not express detectable levels of p11, whereas it is highly expressed in the stromal cells surrounding the tumors. Furthermore, there did not appear to be an induction of p11 in the cancer cells that left the tumor and metastasized to the lungs.

We also examined the lung tumors obtained after tail vein injection of Py8119 cells in p11-WT and p11-KO mice. Surprisingly, we observed that the metastatic foci obtained from the lungs of both genotypes showed strong membranous expression of p11. This was more evident in the p11-KO mice, where expression was restricted to the small tumor nodules and was completely absent in the surrounding lung tissues (Figure 5C). We further evaluated p11 expression in both the whole tumor homogenates and Py8119 cells by immunoblotting (Figure 5D,F). As anticipated, whole tumor homogenates from these groups also showed p11 expression in the PyMT/p11-WT tumors consistent with some expression in the stroma. Unlike the PyMT cancer cells, we observed that Py8119 tumor cells showed robust p11 expression (Figure 5F).

### 2.6. Loss of p11 Does Not Affect Plasminogen Activation (or Plasmin Generation) in PyMT Tumors

We previously observed that plasminogen activation is substantially reduced in p11-depleted cancer cells such as colorectal [14], fibrosarcoma [13], pancreatic [16], and lung [15]. We measured plasmin generation in tumor homogenates and cell lines isolated from PyMT tumors and did not observe any difference (Figure 5E and Figure S5B). However, PyMT tumors from p11-WT and p11-KO mice showed a marked increase in plasminogen activation compared to normal mammary glands (Figure S5C). These data suggest that p11 does not play a significant role in tumor cell plasmin generation in the PyMT mammary tumors.

### 2.7. P11 Regulates Expression of Genes and Cytokines Affecting Tumor Progression

**Figure 6:**
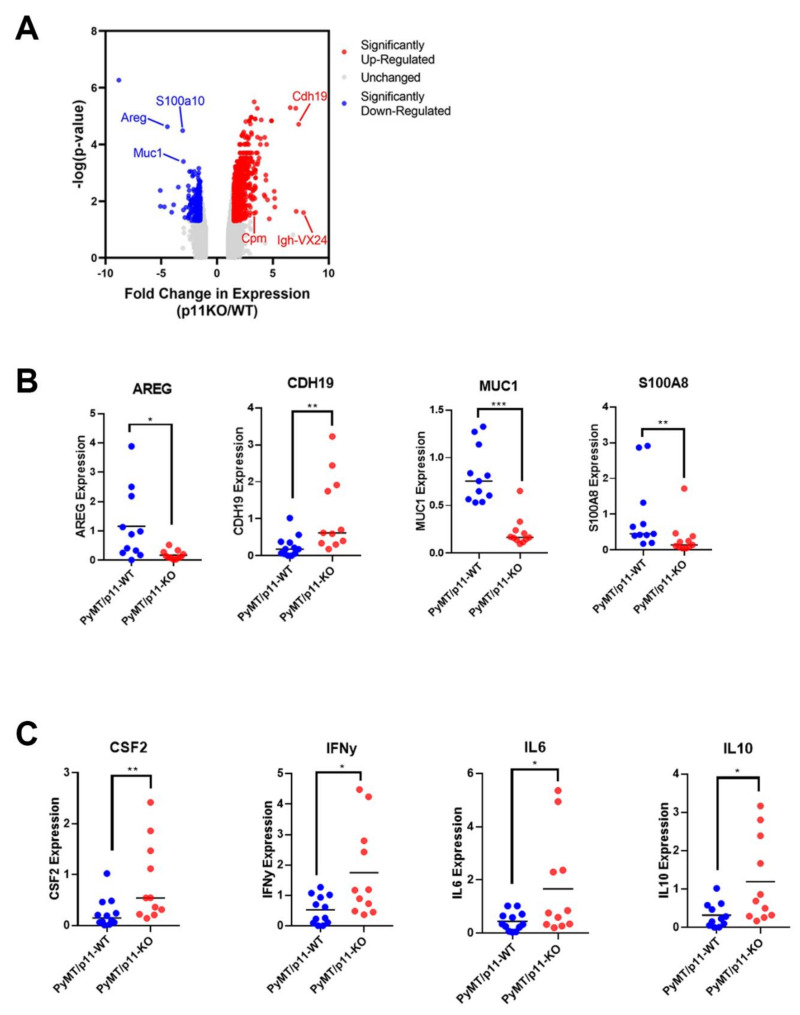
Tumor transcriptome of PyMT/p11-KO mice suggest the downregulation of tumor promoting genes. Gene expression profiling of PyMT/p11-WT and PyMT/p11-KO tumors were performed (n = 3 mice per group). (**A**) Volcano plot with (significantl *p* value of < 0.05) upregulated and downregulated genes in the PyMT/p11 KO mice (n = 3 mice in each group). The top 6 genes are highlighted. Genes which were up- or down-regulated more than 2-fold (log_2_ = 0.678) at a significance level of *p*  < 0.05 were considered differentially expressed. (**B**) Validation of the top 9 differentially expressed genes using quantitative RT-PCR (10–11 mice per group). Significance was determined by unpaired *t*-test. Only the genes significantly altered are shown. (**C**) Loss of p11 in PyMT tumors results in differential cytokine expression profile. We performed quantitative RT-PCR on tumors isolated from PyMT/p11-WT and PyMT/p11KO mice (10–11 mice per group), using a CFX96 or CFX384 Touch Real-Time PCR Detection system (BioRad, Mississauga, ON, Canada). Relative mRNA expression was log-2 transformed prior to plotting and statistical analysis. Significance was determined by unpaired, *t*-test. Only those cytokines significantly altered are shown. The asterix on each plot represents p-values as follows. * *p* < 0.05, ** *p* < 0.01, *** *p* < 0.001.

To examine how p11 might regulate tumor growth and metastasis genes, we performed microarray gene expression profiling on mammary tumors from PyMT/p11-WT and PyMT/p11-KO mice (n = 3 per group). We observed that 891 transcripts were elevated and 269 were reduced by more than 1.5-fold (*p* < 0.05) in the PyMT/p11-KO tumors (Figure 6A). As anticipated, S100A10 expression was decreased by 3.08-fold in the PyMT/p11-KO tumors, supporting the validity of the gene expression data set. Of the 891 transcripts, 331 were annotated genes: 144 genes were downregulated and approximately 187 genes were upregulated in the PyMT/p11-KO mice (Supplemental file 1). We chose to validate seven downregulated genes (*Aldh1a2*, *Areg*, *Ctse*, *Muc1*, *S100a8*, *Thbs1*, and *Tnc*) and two upregulated genes (*Cdh19* and *Cpm*) using reverse transcriptase quantitative PCR (RT-qPCR) (n = 11 mice per group). We confirmed that amphiregulin (Areg), mucin 1 (*Muc1*), and *S100a8*, were downregulated, whereas cadherin 19 (*Cdh 19*) was upregulated by qPCR (Figure 6B). In contrast to the microarray data, *Aldh1A2* and *Tnc* were shown to be upregulated by qPCR (Figure 6B and Figure S6A), which potentially represents false hits from microarray.

Quantitative PCR showed no significant change in the expression of cytokines associated with macrophage recruitment including *Csf1*, *Ccl5*, *Ccr5*, and *Ccl2* (Figure S6B). Comparison of expression of inflammatory cytokines between the two groups showed significant increases in *Ifn*-*γ* (3.36-fold), *Il-10* (3.7-fold), *Il-6* (3.7-fold), but no significant changes in *Il-12α*, *Il-4*, and *Tnf-α* (Figure 6C, Supporting Figure 4B). Interestingly, we observed a 3-fold increase in *Csf2* expression in the PyMT/p11-KO tumors (Figure 6C).

### 2.8. p11 Is Associated with Poor Clinical Outcomes

**Figure 7:**
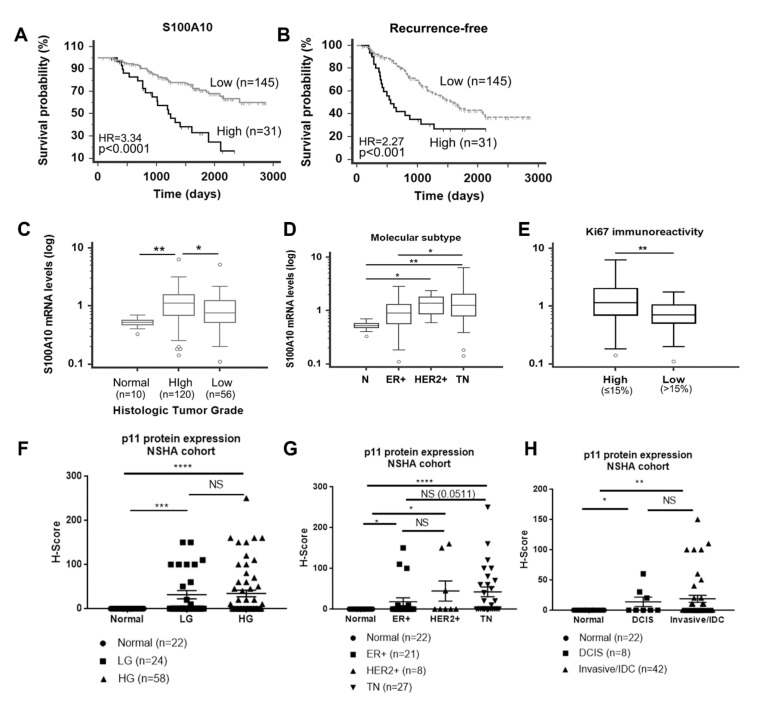
Expression of S100A10 (p11) expression in breast cancer patients. Treatment-naïve primary breast cancer samples (n = 176) were obtained through the Canadian Breast Cancer Foundation (CBCF) Tumor Bank and used for gene expression analysis. *S100A10* high and low mRNA levels were based on the signal intensity from our gene expression microarray profile using the Receiver Operating Characteristic (ROC) curve analysis. Correlation of *S100A10* mRNA with (**A**) overall survival probability. High mRNA levels of *S100A10* are significantly associated with poor patient overall survival (HR of 3.34). (**B**) Recurrence free survival, with HR 2.27. (**C**) For histological tumor grade, p11 is significantly upregulated in high grade (HG) tumors compared to normal breast and low-grade (LG) tumor tissues. (**D**) For molecular subtype, p11 is significantly up-regulated in HER2+ and triple negative breast cancers compared to normal breast tissues. (**E**) In breast cancer patients, p11 mRNA levels are significantly higher in tumors with high Ki67 immunoreactivity. *S100A10* (p11) protein is overexpressed in breast tumors compared to normal mammary tissues. (**F**–**H**) IHC staining of p11 in normal breast tissues and in low grade (LG), high grade (HG), ductal carcinoma in situ (DCIS), IDC(Invasive Ductal Carcinoma)/Invasive, ER+, HER2+ and TN tumors was performed using anti-p11 antibody (Proteintech, Rosemont, IL, USA). These human tissues were obtained from Queen Elizabeth II Health Sciences Centre, Nova Scotia Health Authority (NSHA) (see Materials and Methods). The stained sections were scored (semi-quantitative) based on percent positive tumor cells and intensity of staining by a pathologist (blinded). H score was determined based on the formula described and the samples/tissues were separated and plotted based on (**F**) clinical/histological grade, (**G**) pathological grade, and (**H**) molecular subtype. Statistical analysis was performed by Mann Whitney U test (unpaired, non-parametric test for means *t*-test). The Asterix on each plot represents p-values as follows. * *p* < 0.05, ** *p* < 0.01, *** *p* < 0.001, **** *p* < 0.0001.

We employed a well-defined breast cancer patient cohort (n = 176) and stratified the patient population into S100A10 high and low mRNA levels. Kaplan–Meier survival analysis showed that high levels of S100A10 were significantly associated with both shorter overall survival (HR = 3.34, *p* < 0.0001) and recurrence-free cancer (HR = 2.27, *p* < 0.001) (Figure 7A,B). We further found that S100A10 levels were significantly increased in high histologic grade tumors compared with normal mammary tissues (*p* < 0.01) and low grade tumors (*p* < 0.05) (Figure 7C) Among the molecular subtypes, S100A10 mRNA levels were significantly higher in triple negative (TN) and human epidermal growth factor receptor 2 (HER2)-enriched breast tumors compared to normal breast tissues. TN tumors showed significantly higher S100A10 levels than luminal estrogen receptor (ER +) tumors (Figure 7D). In addition, tumors with high Ki67 immunoreactivity (percentage of positive cells >15%) showed significantly higher S100A10 levels than tumors with low Ki67 immunoreactivity (≤15%) (Figure 7E).

Immunostaining of the NSHA patient cohort followed by semiquantitative scoring of the staining showed no p11 immunostaining (100% negative, H-score = 0) of epithelial cells that formed the normal mammary ducts but expression in the stromal areas surrounding the normal duct was observed (Figure S7A). Based on H-score, we found 25% of low grade, 29.3% of high grade, 37.5% of DCIS, 33% of IDC, 23.8% of ER+, 37.5% HER2+, and 52% of TN tissues/samples were positive for p11 expression. We observed a significant increase in H-score values between normal and low-grade, and normal and high-grade, but the difference between low grade and high grade was not significant (Figure 7F). Similarly, DCIS and IDC samples showed significantly higher H-score values compared to normal tissues. But there was no difference in p11 expression between DCIS and IDC samples, suggesting that p11 expression does not correlate with invasive progression (Figure 7G and Figure S7). We observed the most dramatic increase in p11 staining between normal tissue and TN tumors (*p* < 0.0001), consistent with the highest mean H-score value for the TN tumors. We also noted significant increase between normal and ER+, and normal and HER2+ tumors, but there was no significant increase between ER+ and HER2+ tumors. Interestingly, statistical comparison of ER+ and TN tumors showed increase in TN with respect to ER+ although the p value (0.0511) was only approaching significance (Figure 7H and Figure S7).

## 3. Discussion

The loss of components of the plasmin-plasminogen system decreased pulmonary metastasis but had no effect on tumor onset or growth in the PyMT model (reviewed in [4]). Interestingly, in this study, we observed a dramatic delay in tumor onset, growth, and progression to malignancy in the PyMT/p11KO mice. These results suggest a complex role for p11 in breast cancer oncogenesis beyond the regulation of plasmin generation.

We observed a dramatic decrease in metastatic burden (18-fold) and metastatic foci (14-fold) in PyMT/p11-KO mice. The histopathological progression to late carcinoma stage was delayed in PyMT/p11-KO mice (Figure 2E), potentially attributing the decrease in metastases to the delayed development of malignancy. However, pulmonary metastases in the PyMT model is an early event and independent of tumor size [39,40]. Interestingly, metastases formed by injection of the PyMT transformed cell line, Py8119, into the p11-KO mice were dramatically reduced, suggesting that the p11-deficient stroma is a less favorable environment for the establishment of metastases.

These results are consistent with our previous work showing a dramatic reduction in the growth of Lewis lung carcinomas and T241 fibrosarcomas in p11-deficient mice compared with WT mice [19] and that the tumor growth deficit corresponded with a decrease in macrophage and endothelial cell density. Consistent with delayed tumor onset and growth, we observed a dramatic decrease in Ki67 positivity in tumors from PyMT/p11-KO mice, suggesting a role for p11 in tumor cell proliferation. Although, apoptosis as shown by TUNEL staining was marginally decreased with loss of p11 in PyMT tumors, this was not statistically significant and warrants further testing in a larger sample size. Nevertheless, this corroborates our finding with Ki67 staining/quantification. Tumor-associated macrophages (TAMs) significantly contribute to tumor progression, angiogenic switch, and metastasis [41,42], with loss of peritumoral TAMs resulting in delayed tumor progression [43]. Therefore, it is likely that a portion of the requirement of p11 for PyMT-driven tumor growth and metastasis is due to the function of p11 in macrophage recruitment.

We have previously reported that p11 is responsible for much of the plasmin generation in various cancer cell lines [5,15,16,44]. However, in the present study, we were unable to detect any differences in plasmin generation between cancer cells or tumor homogenates isolated from the PyMT/p11-KO and PyMT/p11-WT mice. Macrophages and endothelial cells utilize p11 for their physiological functions. We have shown that the loss of p11 slows migration of the macrophage to the tumor site. It was therefore surprising that we did not detect differences in the tumor homogenates isolated from PyMT/WT and PyMT/P11KO mice. Although the PyMT/p11-KO mice tumor homogenates appeared to generate less plasmin, the difference did not meet statistical significance. The reason for this discrepancy is unclear at present but may be addressed by larger sample sizes. However, we observed that PyMT tumors from p11-WT and p11-KO mice showed a marked increase in plasmin generation compared to normal mammary glands (Figure S5D), suggesting compensation by other plasminogen receptors in this model. It is likely that distant peripheral macrophages utilize plasminogen receptors such as p11 to migrate and invade the tumors [19,45,46]. Furthermore, we detected weak p11 expression in some of the tumor cells and no expression in metastatic nodules of PyMT/p11-WT tumors. The similarity of F4/80 and p11 staining pattern in the PyMT-tumor stroma suggests that macrophages are the most prominent p11-staining cells in the stroma (Figure S8). However, detailed colocalization studies will be necessary to verify this prediction. In contrast, lung tumors obtained after tail vein injection of Py8119 cells in p11-WT and p11-KO mice showed robust p11 expression. Overall, the exclusive expression of p11 in the stroma in vivo parallels the stromal localization of other components of the plasminogen activation system including uPA, uPAR, tPA, and PAI-1 in mouse (PyMT) and human models [4]. The lack of p11 expression in the PyMT-expressing tumor cells and restriction of the majority of p11 expression to the stromal compartment were striking observations in the current study. The middle T (MT) region of PyMT is an effective oncogene and activates the PI3 kinase (PI3K) signaling pathway [47,48]. Recently we have shown that active PI3K signaling decreased p11 expression in several tumor cells via the FOXC2 transcription factor [15]. In addition, *in vitro* observations indicate a distinct rewiring of signaling in 2-D cell cultures resulting in increased p11 expression potentially due to de-repression of tumor-stroma signaling events observed in vivo [49,50]. Furthermore, the retention of p11 expression in the Py8119 tumor cells in the lung metastatic nodules, suggests the absence of inhibitory signals in the lung microenvironment.

Whole mount analysis of mammary glands indicated a decrease in multifocal dysplastic lesions as early as 6–8 weeks in the PyMT/p11-KO mice (Figure 1). By 10–12 weeks, these lesions had become quite dramatic, whereas dysplastic lesions in the mammary glands of the PyMT/p11-KO mice were sparse. An interesting observation was the reduction in length of the ductal branches in the PyMT/p11-KO mammary glands at the early time points of 6 and 8 weeks, and was comparable to the WT at 10 and 12 weeks (Figure S2). However, by 10 and 12 weeks, the ductal branching was indistinguishable, suggesting that the developmental deficit was no longer present (Figure S2). The mechanism by which the developmental deficit had been overcome at 10 weeks is unclear and will require a detailed study of the role of p11 in mammary development. However, it is unclear if this novel function of p11 in mammary development could account for the dramatic reduction in tumor growth observed in the PyMT/p11-KO mice. Other groups have shown that infiltration of macrophages is required for early mammary gland development [51]. It is likely that defective macrophage migration observed with p11-KO mice causes early defects in ductal branching in the mammary gland. This suggests that p11 may play a potential role during early stages of mammary gland development. More recently, another plasminogen receptor, Plg-Rkt, was shown to be critical for lactogenesis and mammary lobuloalveolar development [52]. We were initially concerned that the delayed development might affect the expression of the PyMT oncogene in the epithelial cells and thereby contribute to the delay in oncogenesis observed in the p11-KO mice. However, we did not observe a decrease in PyMT expression in spontaneous tumors from p11-KO mice as evidenced from both qPCR and IHC expression analysis at several early and late time points (data not shown). This suggests that delay in development does not affect oncogene expression in p11-KO mice and therefore the effects of p11 on tumor development are direct. These data and the evidence from human patient tumor analysis strengthens our hypothesis that p11 plays a development-independent role in tumor growth and progression. Our future studies are focused on investigating the role of p11 in mammary gland development.

Quantitative PCR validation of microarray gene data confirmed the significant downregulation of *Areg*, *Muc1*, and *S100a8* genes in PyMT/p11-KO tumors. *Areg* (amphiregulin), a player in breast cancer proliferation, is abundant in the pubertal mammary gland and loss of *Areg* in mice results in stunted ductal morphogenesis. Our observation of decreased early mammary ductal morphogenesis might be due to the loss of *Areg* expression [53]. Loss of transmembrane glycoprotein *Muc1* results in significant delay in tumor progression and metastasis [54], consistent with delayed progression to malignancy displayed by PyMT/p11-KO tumors. *S100a8* is elevated in ER- and HER2+ subtypes of breast cancer and contributes towards cancer cell survival and metastasis [55], thereby contributing to delayed progression of PyMT/p11-KO tumors. It is unclear whether p11 plays a direct or indirect role in regulating these genes in vivo, or whether the alteration of these genes are markers of tumor growth and progression.

We also compared the levels of inflammatory modulators in PyMT/p11WT and PyMT/p11-KO tumors that might impact macrophage infiltration. The *Csf1*- gene, implicated in the proliferation, differentiation, and recruitment of macrophages in breast cancer [37], was unchanged. We saw a significant increase in *Csf2* expression in PyMT/p11-KO tumors but *Csf2* has not been shown to correlate with macrophage infiltration in breast tumors. We also observed a significant increase in cytokines such as *Ifn-γ*, *Il-6*, and *Il-10*. *Il-6* positively correlates with breast cancer progression and development of metastasis [56]. Studies on the role of *Il-10* in breast cancer showed contradicting results [56]. *Ifn-γ* plays an important role in antitumor immunity and tumor suppressive phenotype via the JAK/STAT pathway. It is possible that the tumor suppressive phenotype observed in the PyMT/p11-KO mice is ascribed to the increase in tumor suppressive cytokines such as *Il-10* and *Ifn-γ*.

Previous gene expression studies showed that p11 correlated to poor overall survival in basal-like breast cancer (reviewed in [57]. In elucidating the prognostic role of p11 in human breast cancer, we found that p11 mRNA (microarray) was overexpressed in human breast tumors, correlated positively with overall survival and recurrence-free survival, and was increased in high grade tumors compared to low grade tumors and normal tissue. P11 was highly expressed in TN breast cancer, ER+, and HER2+, suggesting a role in breast cancer progression. Interestingly, expression of p11 protein (IHC) was significantly elevated in tumor cells compared to normal mammary epithelium, but we did not observe any correlation between high p11 expression and clinical and pathological tumor grade or with molecular subtype in human breast cancer samples. The lack of consistency between our gene expression profiling and IHC data could be due to two reasons. First, microarray analysis was performed in tumors containing stroma and hence the increased p11 expression includes contribution from the tumor microenvironment (Figure 7A–E). Second, we conducted p11 immunostaining only in a small cohort of patient samples with a small sample size for ER+ and HER2+ tumors (Figure 7F–H and Figure S7), where the H-scores were ascribed based on the staining intensity in the tumor cells. Future studies will be aimed at increasing the sample size for IHC or by using a tissue microarray analysis and evaluating the expression pattern in the stroma. Overall, p11 expression was completely restricted to the stromal cells in the PyMT mouse model, whereas expression was seen in human tumor cells. This difference can potentially be attributed to the nature of oncogene and mutations between the murine and human breast tumors.

## 4. Materials and Methods

### 4.1. Mice

All animal experiments were performed according to protocol approved by the University Committee on Laboratory Animals, Dalhousie University, Canada (Protocol # 18-039). The C57Bl6 MMTV-PyMT mice were obtained from Dr. Mak (UHN, Toronto, Canada). The p11 WT and p11 KO mice were obtained from P. Svenningsson (Karolinska Institutet, Solna, Sweden). We employed a sequential breeding strategy to generate PyMT/p11-KO homozygous mice. We crossed the male PyMT/p11-WT mice with the female p11 KO mice to obtain heterozygotes for p11. The resultant F1 (PyMT/p11+/−) males were bred with the female (p11+/−) mice. This crossbreeding produced F2 females PyMT/p11-WT, (PyMT/p11+/−) and PyMT/p11-KO. Virgin female C57BL/6 mice heterozygous for the MMTV-PyMT transgene and homozygous KO (PyMT/p11-KO) or homozygous WT (PyMT/p11-WT) for the p11 gene were used in our studies. Genotyping of mice was performed by PCR of genomic DNA derived from ear clippings using published primers for PyMT and p11 (Table S1).

### 4.2. Cell Culture and Reagents

Py8119 cells were obtained from American Type Culture Collection (ATCC) and maintained in F12 Kaign’s medium (Hyclone, Logan, UT, USA) with 5% Fetal Clone II serum (Thermofisher Scientific, Whaltham, MA, USA), 1% penicillin and streptomycin. Py8119 cells were tested for pathogen (Charles River Laboratory, Wilmington, MA, USA) before injecting into the mice. All cells were tested for mycoplasma (Lonza Mycoalert kit, Morristown, NJ, USA) and only mycoplasma-free cells were used.

### 4.3. Whole Mount Analysis

The harvested tissues were stretched and placed onto a cassette matrix and fixed overnight in Carnoy’s fixative. The tissues were sequentially hydrated with 70%, 50%, 25% ethanol, followed by a final water wash. They were stained with carmine alum solution for four hours, followed by sequential dehydration in 70%, 95%, and 100% ethanol, and defatting of the tissues was carried out in two xylene washes for 30 min and overnight. The dehydrated and defatted tissues were then mounted on slides using Cytoseal (Richard-Allan Scientific, Thermo Scientific, Waltham, MA, USA) mounting medium. The slides were digitally imaged using a stereomicroscope under similar lighting and contrast conditions (Zeiss microscope).

### 4.4. Tumor Measurements

For the spontaneous PyMT tumor model, all the mammary glands were palpated once a week for 20–25 weeks to monitor tumor latency and progression. For evaluating the kinetics of tumor growth, the tumors were measured using Vernier calipers, and tumor volume was determined using the standard calculation for a hemi-ellipsoid; 0.5ab^2^, where a is the smaller and b is the larger diameter. Tumor burden was calculated at the endpoint (20 weeks) by determining the total tumor weight/body weight. We determined the percentage of tumor-free mice using Kaplan–Meier analysis (Graph pad prism, La Jolla, CA, USA) in the two PyMT mice groups. Animals were considered tumor-free until a palpable mass (>4.0 mm) persisted for longer than four days. All tumor growth and kinetics data were obtained from three independent experiments. Only virgin female mice were used for all the experiments.

### 4.5. Spontaneous and Experimental Metastasis Quantification

For evaluation and assessment of lung metastasis, lungs were harvested at 20 weeks, fixed in 10% buffered formalin for 48 h, and embedded in paraffin. The lungs were cut at 5 µm thickness and 3–6 sections from each lung at 100 µm apart were stained with H&E. The stained slides were scanned using Aperio microscope (Leica Biosystems, Concord, ON, Canada) and the number of metastatic foci were counted using Imagescope (Leica Biosystems, Concord, ON, Canada). The total metastatic burden was determined using Imagescope by tracing the metastatic area as a ratio of the total lung area. For experimental metastasis assay, Py8119 cells were injected via the tail vein at a density of 2.5 × 10^5^ cells. The lungs were harvested 14 days after injection and analyzed for metastatic burden and foci calculation as described for spontaneous metastasis quantification.

### 4.6. Tissue Processing and Immunohistochemistry

Tumors, mammary glands, and lungs were harvested and fixed in neutral 10% buffered formalin for 48 h and stored in 70% ethanol before embedding in paraffin. The embedded tissues were cut into 5 µm thick sections, stained with H&E for pathological analysis, and adjacent sections were used for IHC. The tissue sections were deparaffinized with sequential washes in xylene, 100%, 95%, and 70% ethanol followed by water wash. The details of the antibody dilution, method of chromogen, and stain development are provided in the Table S2.

Antigen retrieval was performed by heat treatment using a pressure cooker in either Tris-EDTA (pH 9.0) or citrate buffer (pH 6.0). The slides were rinsed in Tis-buffered-saline (TBS) or Phosphate Buffered Saline (PBS), depending on the antibody and blocked with Rodent Block M (Biocare, Pacheco, CA, USA). The primary antibodies CD31, Ki67, smooth muscle α-actin (α-SMA), p11, F4/80, were incubated overnight at room temperature followed by secondary antibody and chromogen steps. For anti-rat, the slides were washed with TBS after primary incubation, followed by 15 min incubation with rat probe, subsequently the slides were washed and incubated with Rat on mouse HRP polymer for 15 min (Biocare, Pacheco, CA, USA), followed by three washes with TBS and stained with DAB (Biocare, Pacheco, CA, USA) and counter stained with hematoxylin. For anti-rabbit secondary antibodies, the slides were incubated with goat anti-rabbit secondary (Envison, DAKO, Beijing, China) for 30 min, followed by washes with TBS and counterstained with hematoxylin. For anti-mouse antibody, we used mouse-on-mouse HRP polymer kit (Biocare, Pacheco, CA, USA) and anti-goat antibodies we used rabbit-anti goat HRP polymer (DAKO), followed by staining with DAB and counterstaining with hematoxylin. All stained sections were washed in water and mounted with aqueous mounting medium (Vectamount, MJS Biolynx Inc., Brockville, ON, Canada). The immunostained sections were imaged using Zeiss Axio Imager Z1 W/color and monochrome camera (p11, Ki67, CD31, F4/80, α-SMA, H&E, CD3, and TUNEL staining) using 40X (for manual counting–F4/80, Ki67, CD3) and 20X for representation. To count the number of CD31 positive vessels, we scanned the slides using Aperio Scanning system, following which CD31-positive vessels were counted from 7–10 snapshots of random fields. To quantify the number of Ki67 positive, F4/80, CD3-positive, TUNEL positive, we captured images using Zeiss Axio Imager Z1 microscope, and manually counted the positive cells using the Zen software in 7–10 fields. The average of 7–10 fields for Ki67, CD31, and F4/80, was used for each mouse in the quantification bar graph. For human p11 staining, we followed the same procedure as for mouse tissues, but blocking was performed with Background Terminator (Biocare, Pacheco, CA, USA) for 10 min before incubating with primary antibody.

### 4.7. Gene Expression Profiling (Mouse Tumors)

Sample preparation, amplification, and hybridization to the Affymetrix Mouse gene 2.0 ST array, and data collection were performed by The Centre for Applied Genomics at the Hospital for Sick Children (Toronto, ON, Canada). The Transcriptome Analysis Console software (Thermo Fisher Scientific, Waltham, MA, USA) was used to normalize data and calculate fold changes in expression (GSE151579). Genes up- or downregulated by more than 2-fold (log_2_ = 0.678) at a significance level of *p* < 0.05 were considered differentially expressed.

### 4.8. Gene Expression Profiling (Human Tumors)

The gene expression microarray dataset was generated from a human breast cancer cohort consisting of 176 treatment-naïve primary tumor samples as previously described [58] (GSE22820). Patient material and clinical information were collected under Research Ethics Board Protocol ETH-02-86-17. Patients received standardized guideline-based chemo and hormone therapies: i.e., hormone therapy for all patients with ER-positive tumors, trastuzumab for those with HER2-overexpression tumors, anthracycline chemotherapy for high risk node-negative disease, and anthracycline plus taxane chemotherapy for node-positive disease. The median follow-up time for surviving patients was 4.5 years.

### 4.9. Quantitative Polymerase Chain Reaction (Mouse Tumors)

Tumors from PyMT/p11-WT and PyMT/p11-KO mice at 20 weeks of age were excised, snap frozen in liquid nitrogen, and stored at −80 °C. Total RNA was extracted using Trizol (Invitrogen, Thermofisher Scientific, Waltham, MA, USA) and the PureLink RNA kit (Invitrogen, Thermofisher Scientific, Waltham, MA, USA ) with DNase treatment. Equal amounts of RNA (0.5 µg) were reverse transcribed using iScript (BioRad, Mississauga, ON, Canada); qPCR reactions were performed with SsoAdvanced Universal SYBR Supermix (BioRad, Mississauga, ON, Canada) and gene-specific mouse primers (Table S2) on a CFX96 or CFX384 Touch Real-Time PCR Detection system (BioRad, Mississauga, ON, Canada). Standard curves for each primer set were generated, and primer efficiencies were incorporated into the CFX Manager software (BioRad, Mississauga, ON, Canada). mRNA expression of all samples was calculated using the ΔΔct method, with gene of interest made relative to two reference genes (rlp10 and PyMT) and an indicated control sample. Relative mRNA expression was log−2 transformed prior to plotting and statistical analysis.

### 4.10. Human Patient Breast Tissue Collection, p11 Immunohistochemistry, and H-Score Assignment

Anatomical pathology electronic files (Cerner Millennium) for the Queen Elizabeth II Health Sciences Centre, Nova Scotia Health Authority (NSHA) were retrospectively searched for a cohort of invasive and in situ breast carcinomas beginning 1 January 2011. One formalin-fixed paraffin-embedded (FFPE) tumor block and one FFPE block of normal breast tissue were selected from each patient of an identified cohort of 119 patients. For this study, FFPE breast tissues were used with approval from the Nova Scotia Health Authority Research Ethics Board, and Materials Transfer and Collaboration Agreement between the NSHA and Dalhousie University. P11 immunostaining on patient tissue sections was performed as described earlier [16], using anti-rabbit polyclonal antibody to human p11 (Proteintech, Rosemont, IL, USA).

The stained sections were evaluated and assigned a semiquantitative score by a pathologist (PJB) in a blinded fashion based on percentage of positive cells and intensity of staining. The percentage of cells were identified as negative, weak, moderate, and strong for membrane staining in tumor cells. An H-score of less than 10% was considered negative. The H-score was determined using the formula (% negative × 0 + % weak × 1 + % moderate × 2 + % strong × 3). The semiquantitative H-score values ranged at 5–250. Nottingham grade 1 and 2 tumors were combined as ‘low grade’ and Nottingham grade 3 tumors were labeled ‘high grade’. A scatter plot was generated based on tumor grade (high and low grade, including samples from the molecular subtype category), tumor type (ductal carcinoma in situ (DCIS), variants of invasive ductal carcinoma and invasive lobular carcinoma, also including samples from the molecular subtype categories), and molecular subtype (estrogen receptor positive (ER+), Her2+, triple negative (TN).

### 4.11. Isolation of Mouse PyMT Tumor Cell Lines

PyMT tumors from 20-week mice were excised and rinsed in (PBS), minced in RPMI with 5% FBS with penicillin and streptomycin containing 1 mg/mL of collagenase (Sigma, Oakville, Ontario, Canada). The tumor preparation was then incubated at 37 °C for 1–2 h, followed by straining through a 70-micron mesh strainer. The cells were washed with Dulbecco’s Modified Eagle’s Medium (DMEM) with 10% FBS, passaged once and used in plasmin generation assays as described below. In some cases, isolated cells were directly plated in 96-well plate without passaging and plasmin generation assay was performed as described.

### 4.12. Plasmin Generation Assay

Plasmin generation assay was determined in PyMT tumor homogenates. We employed fresh and frozen 20-week old PyMT tumors from PyMT/p11-WT and PyMT/p11-KO mice to prepare tumor homogenates. Briefly the tumors were homogenized in electric homogenizer (Pro Scientific, Oxford, CT, USA) in Dulbecco’s phosphate-buffered saline (DPBS) with 1% Triton. We used 30–60 µg of protein for the assay. Plasmin generation assay was conducted as described in [19,44].

### 4.13. Immunoblotting

Immunoblotting was performed as described in [59] and anti-mouse p11 antibody (R&D systems, Minneapolis, MN, USA) and β-tubulin (Sigma, Mississauga, ON, Canada) was used for immune staining (details in Table S2).

### 4.14. Statistical Analysis

Three independent experiments with varying mice numbers in each were performed. For evaluation of total palpable tumors (tumor onset), tumor-free mice (survival), tumor growth (volume) and metastasis, we pooled the data/measurements from the three independent experiments. For determination of end-point tumor and metastatic burden and foci, we used data from one independent experiment (20-week endpoint). For time course measurements (whole mounts, tumor progression), we employed 3–12 mice at each endpoint. All statistical analyses were performed using Graph pad prism 5 software (La Jolla, CA, USA). Unless indicated in the figure legends, statistical significance was determined using the Mann Whitney non-parametric test for significantly different variances. * *p* < 0.05, ** *p* < 0.01, *** *p* < 0.001, **** *p* < 0.0001. Proportions were compared by χ^2^ test. A significance threshold of *p* < 0.05 was used.

For human patient samples, all statistical analyses for gene expression profiling were performed using MedCalc Version 14.12.0 (MedCalc Software). Gene expression microarray signal intensity values were log-transformed to better fit the normal distribution assumption. One-way ANOVA was employed to test the statistical significance for the difference in S100A10 mRNA levels among molecular subtypes, tumor histologic grades, or Ki67 immunoreactivity classifications. Prognostic significance was analyzed using log rank test on Kaplan–Meier survival probabilities.

## 5. Conclusions

Our current study is the first comprehensive study using a transgenic mouse model to examine the role of p11 in breast cancer. The novel finding of this study is that p11 potentially plays a role in mammary gland development and future studies are aimed at delineating the mechanistic role of p11 in ductal branching morphogenesis and mammary gland development. Overall, our studies demonstrated that p11 plays a causal, complex, and definitive role in breast tumor development, progression, and metastasis, possibly via p11-dependent macrophage migration and tumor infiltration and regulation of tumor associated cytokines and genes involved in tumor progression. Surprisingly, our results suggest that stromal and not cancer cell p11 is crucial for mediating these effects specifically in the PyMT model. Since the majority of p11 was expressed by the stromal cells in the tumor periphery, our study also highlights the importance of tumor–stroma interactions and signaling for breast tumor progression. Further studies are required to conclusively demonstrate that p11 can be employed as a biomarker with diagnostic and prognostic value in breast cancer.

## Supplementary Materials

The following are available online at https://www.mdpi.com/2072-6694/12/12/3673/s1, Figure S1: Loss of p11 affects multi-focal dysplastic lesions. Figure S2: Loss of p11 affects early stage mammary gland development. Figure S3: Loss of p11 delays progression to late carcinoma–H&E and α-smooth muscle actin IHC. Figure S4: Loss of p11 does not impact cell-death/apoptosis and T-cell infiltration. Figure S5: Loss of p11 does not decrease plasmin generation in PyMT tumors and cell lines. Figure S6: Gene expression profiling of PyMT/p11-WT and PyMT/p11-KO tumors. Figure S7: IHC staining of p11 in human tissue sections. Figure S8: IHC staining of p11 and F4/80 in the PyMT/p11-WT tumors. Table S1: Sequences of primers employed in the q-PCR validation of microarray hits, q-PCR expression of cytokine in PyMT tumors and genotyping for PyMT and p11. Table S2: Antibodies used for immunoblotting and IHC. S1 File. Microarray hits for PyMT mouse tumor gene expression profiling. This file in an excel spread sheet listing all the hits obtained from gene expression profiling of PyMT tumors.
