# Supplementary material for: S100A10 Has a Critical Regulatory Function in Mammary Tumor Growth and Metastasis: Insights Using MMTV-PyMT Oncomice and Clinical Patient Sample Analysis
Source: Cancers (Basel). 2020 Dec 7;12(12):3673. doi: 10.3390/cancers12123673 (PMC7762402; doi:10.3390/cancers12123673)
Supplement: Supplementary file 1 [file cancers-12-03673-s001.zip › cancers-968513_supplementary/cancers-968513_supplementary_conversion.docx]

Supplementary figures and tables


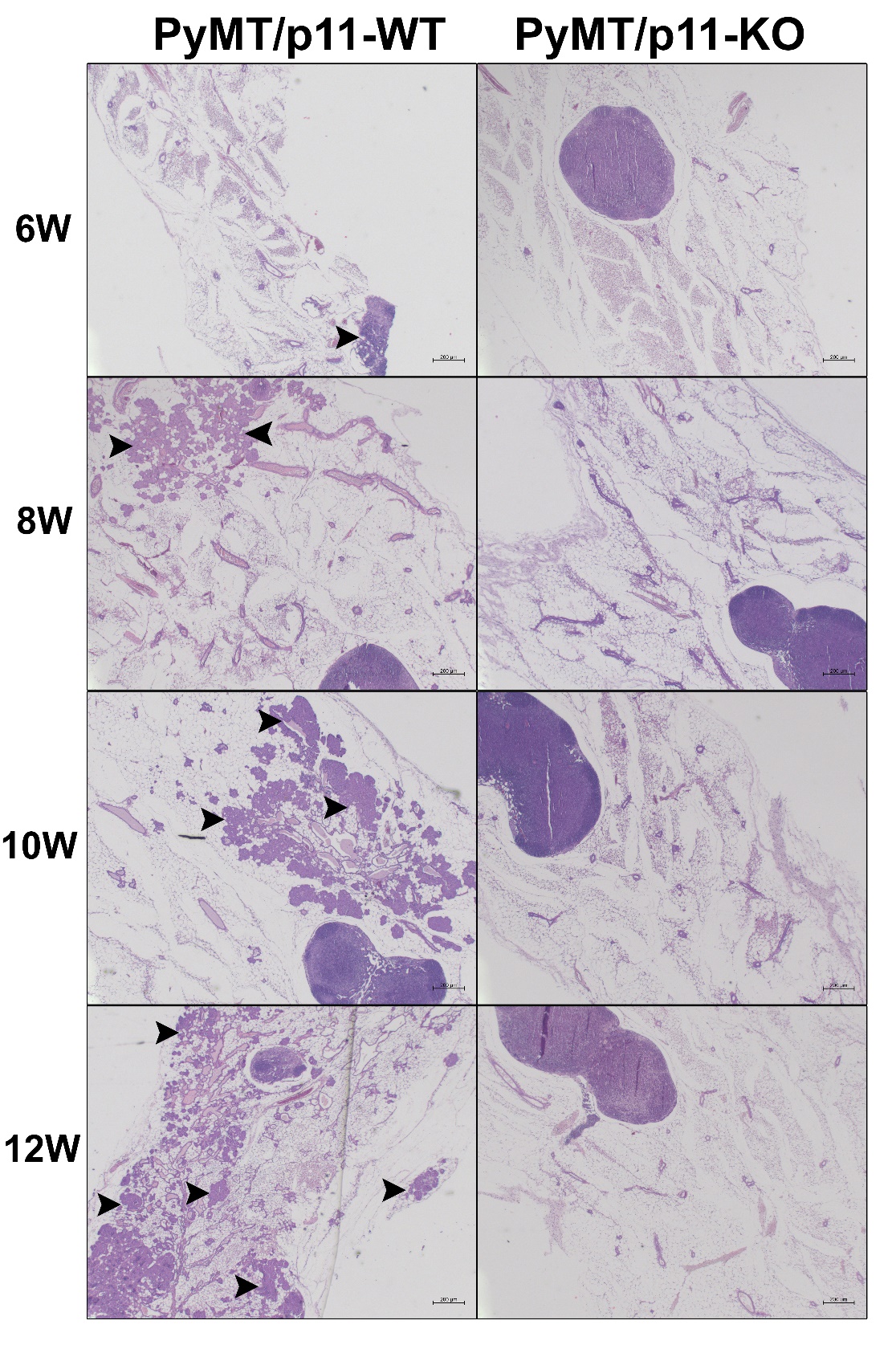


**Figure S1.** Loss of p11 affects multi-focal dysplastic lesions: This figure is supplemental to Figure 1. Representative 5 μM sections of mammary glands from 6 weeks (6W), 8 weeks (8W), 10 weeks (10W), and 12 weeks (12W) mice were stained with Hematoxylin and Eosin and imaged at 5X. Blackhead arrows depict the dysplastic lesions. Scale bar 200µm.


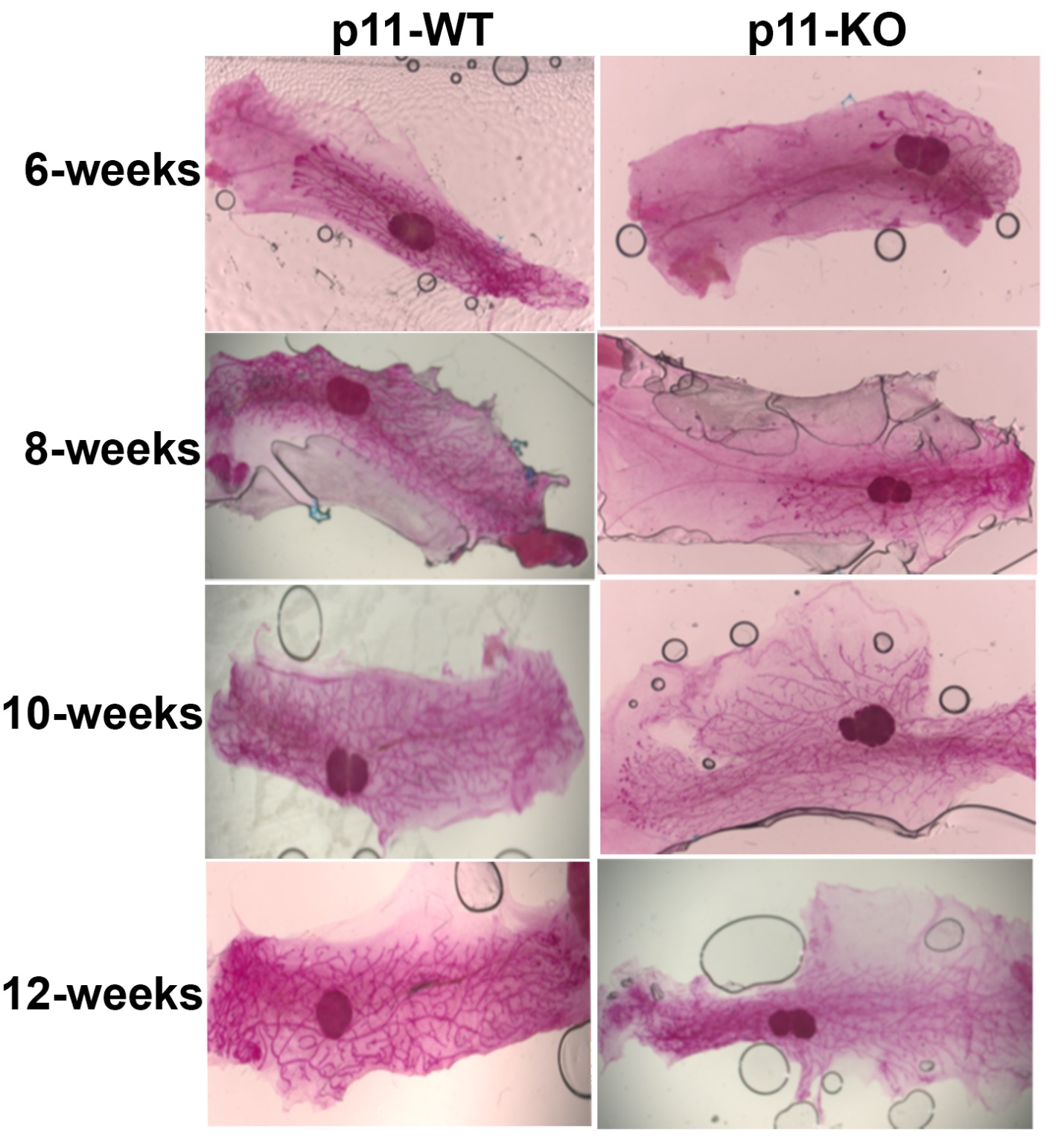


**Figure S2.** Loss of p11 affects early-stage mammary gland development: Whole mammary glands (6, 8, 10, 12 weeks) were excised from p11-WT (n=3), p11-KO (n=3), and stained with Carmine Alum as per standard protocols. Representative images from the 4^th^ abdominal mammary gland from each group and time point are shown.


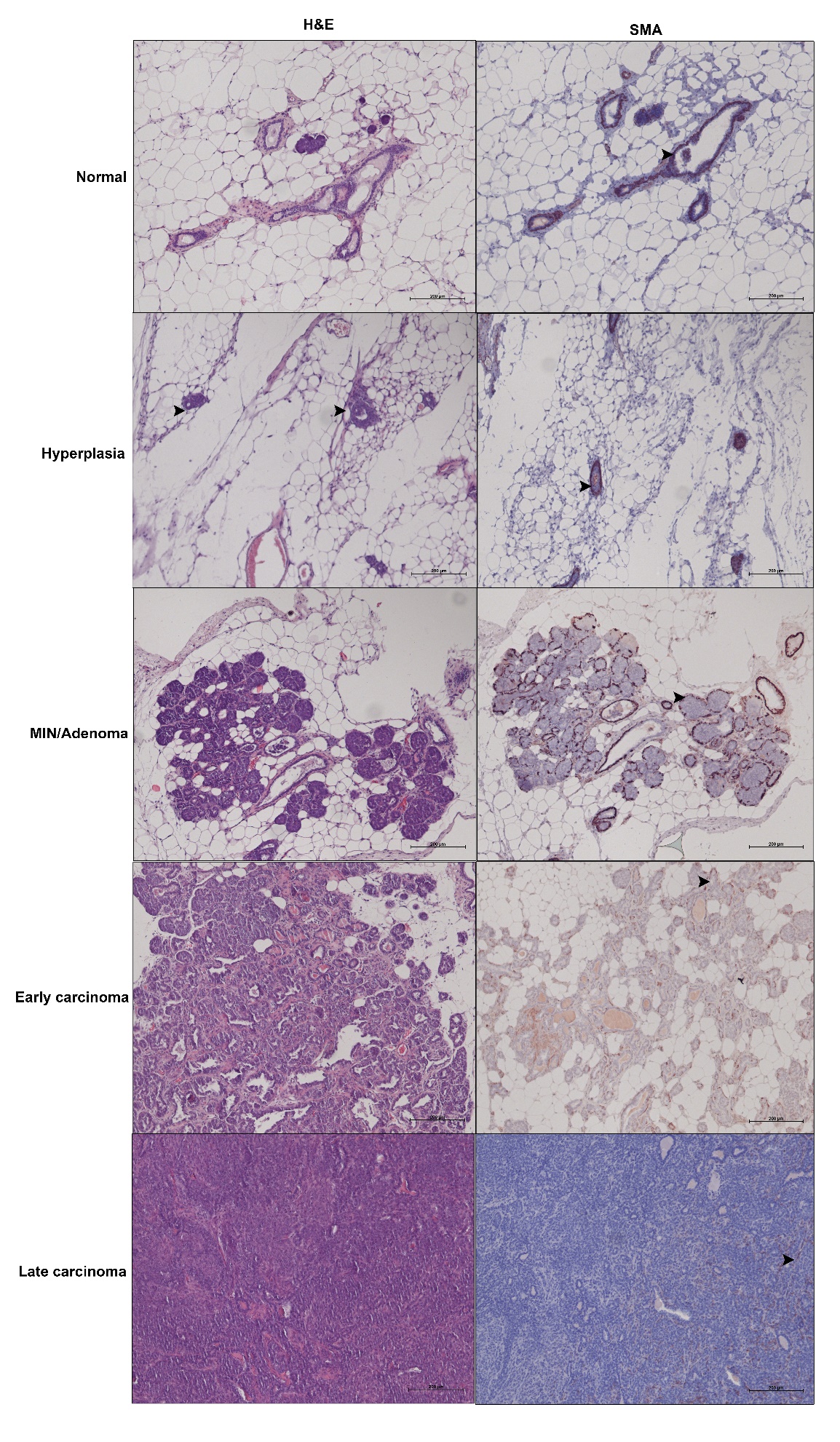


**Figure S3.** Loss of p11 delays progression to late carcinoma – H&E and α-smooth muscle actin IHC. This figure is supplementary to Fig 2 F. Representative 5 μM sections of mammary glands from 10, 12- and 20-weeks mice were stained with Hematoxylin and Eosin (H&E) and α-smooth muscle actin as described in methods and Supplement Table 2. Representative tissues at every stage of progression are shown as normal, hyperplasia, MIN/Adenoma, early and late carcinoma. and imaged at 10X. Blackhead arrows in both panels depict the lesion and corresponding α-SMA staining. Scale bar 200µm.


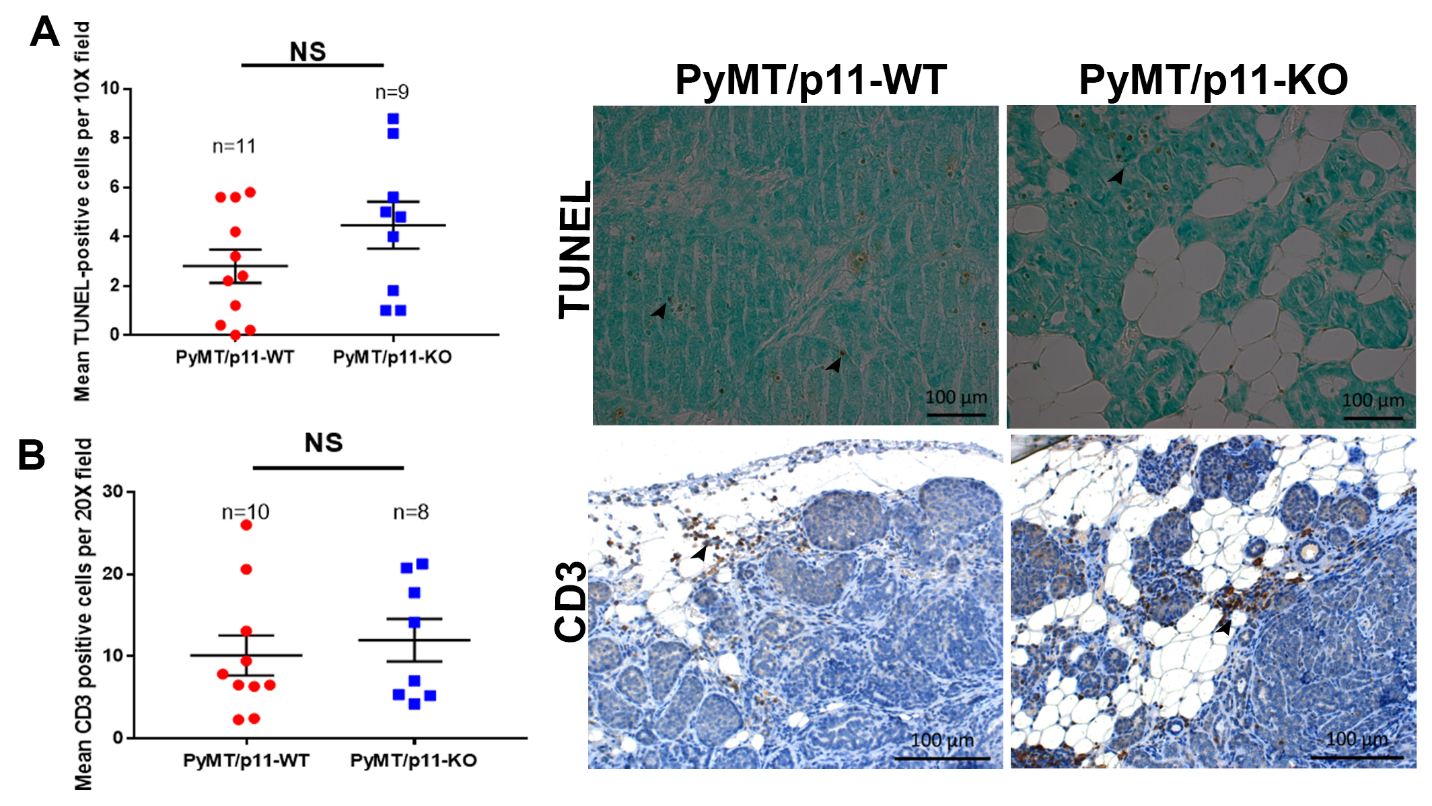


**Figure S4.** Loss of p11 does not impact cell-death/apoptosis and T-cell infiltration: (A) TUNEL staining was performed on formalin-fixed, paraffin-embedded and sectioned tissues from PyMT/p11-WT (n=11) and PyMT/p11-KO (n = 9) (endpoint,20 weeks) using manufacturer’s instructions (Abcam, Cambridge,MA, USA). The stained sections were imaged using Zeiss Axio Imager Z1 W/ color camera (Carl Zeiss Canada, Toronto, ON, Canada) at 10X magnification (*Left panel*) the number of TUNEL-positive cells was manually counted using Zen software. The representative (*Right panel*) image was captured using Zeiss Axio Imager Z1 W/ color camera at 20X magnification. The significance was determined using the Mann Whitney U test (unpaired, non-parametric test for **means**, *p =* 0.2849). Scale bar - 100µm. (B) Formalin-fixed, paraffin-embedded and sectioned tissues from PyMT/p11-WT (n=10) and PyMT/p11-KO (n = 8) (end-point, 20 weeks) mice were stained for CD3 (total T cell marker) using anti-rabbit CD3 antibody (Abcam). The stained sections were imaged using Zeiss Axio Imager Z1 W/ color camera at 10X magnification (*Left panel*) the number of CD3-positive cells was manually counted using Zen 2012 software. The representative (*Right panel*) image was captured using Zeiss Axio Imager Z1 W/ color camera at 20X magnification. The significance was determined using the Mann Whitney U test (unpaired, non-parametric test for means), *p =* 0.6796). Scale bar - 100µm.


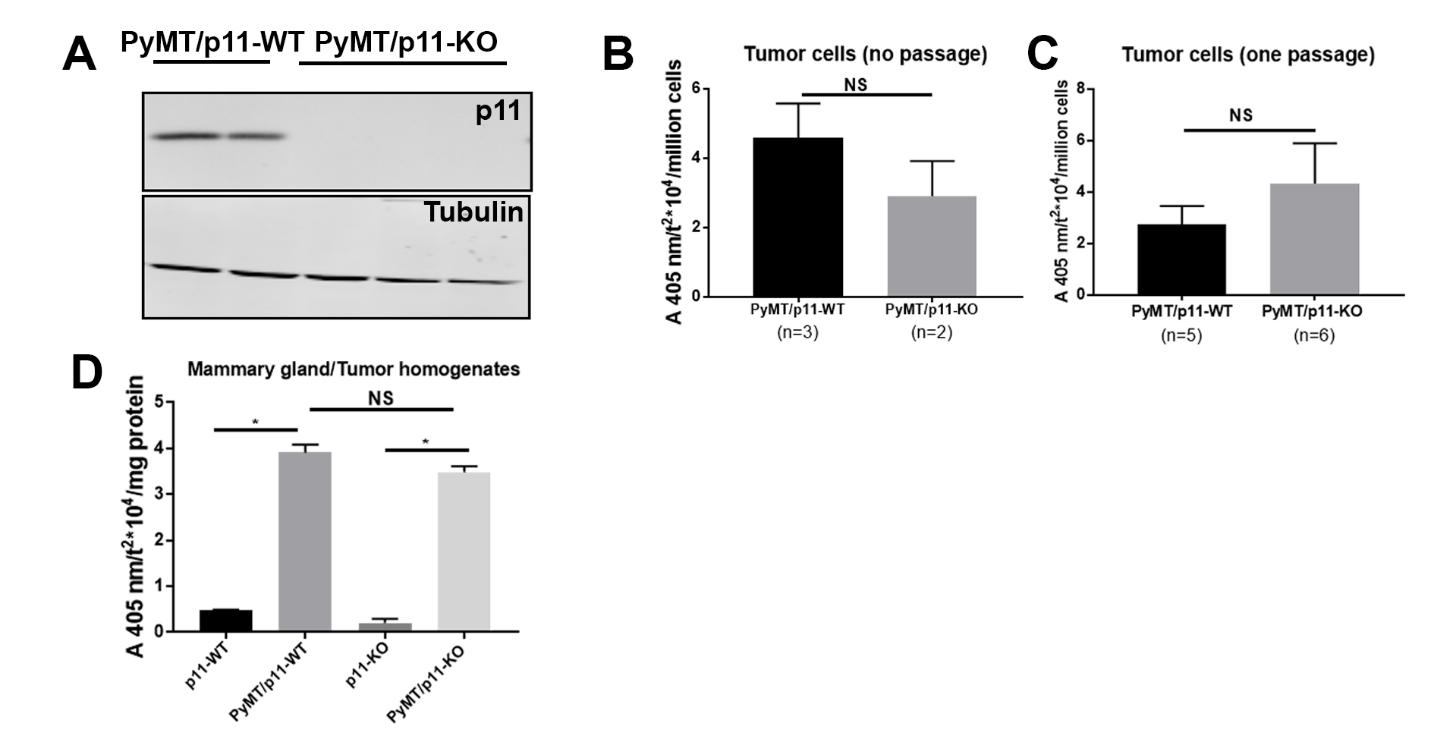


**Figure S5.** Loss of p11 does not decrease plasmin generation in PyMT tumors and cell lines: (A) P11 expression - we isolated tumor cell lines from PyMT/p1l-WT and PyMT/p11-KO mice. 20-25 µg of total protein was separated using 10-20% gradient SDS-PAGE, transferred to nitrocellulose, and probed with p11 antibody (R&D Systems, Minneapolis, MN, USA) and β-tubulin antibody (Sigma, Oakville, ON, Canada). Plasmin generation assay was conducted as described in (39) (19) in cell lines isolated from PyMT mice. Briefly, a total of 5x10^4^ cells PyMT/p11-WT and PyMT/p11-KO tumor cells isolated and either plated directly after isolation in 96-well plate (B) without passaging or (C) passaged once and plated in 96-well plates for 24-hours. Cells were washed and incubated with or without plasminogen (0.5µM – Molecular Innovations, Novi, MI, USA) for 30 minutes at room temperature, followed by the addition of S2251, a chromogenic plasmin substrate (500 mM, Chromogenix, Diapharma Group, West Chester, OH, USA). The rate of appearance of the S2251 cleavage product was measured spectrophotometrically at 405 nm with readings taken every two minutes for 2 hours. (D) 4^th^ inguinal mammary glands from p11-WT and p11-KO and tumors from PyMT/p11-WT and PyMT/p11-KO mice (n=1) was isolated, homogenized as described in materials and methods and 30-60 µg was used for plasmin generation assay as described above.


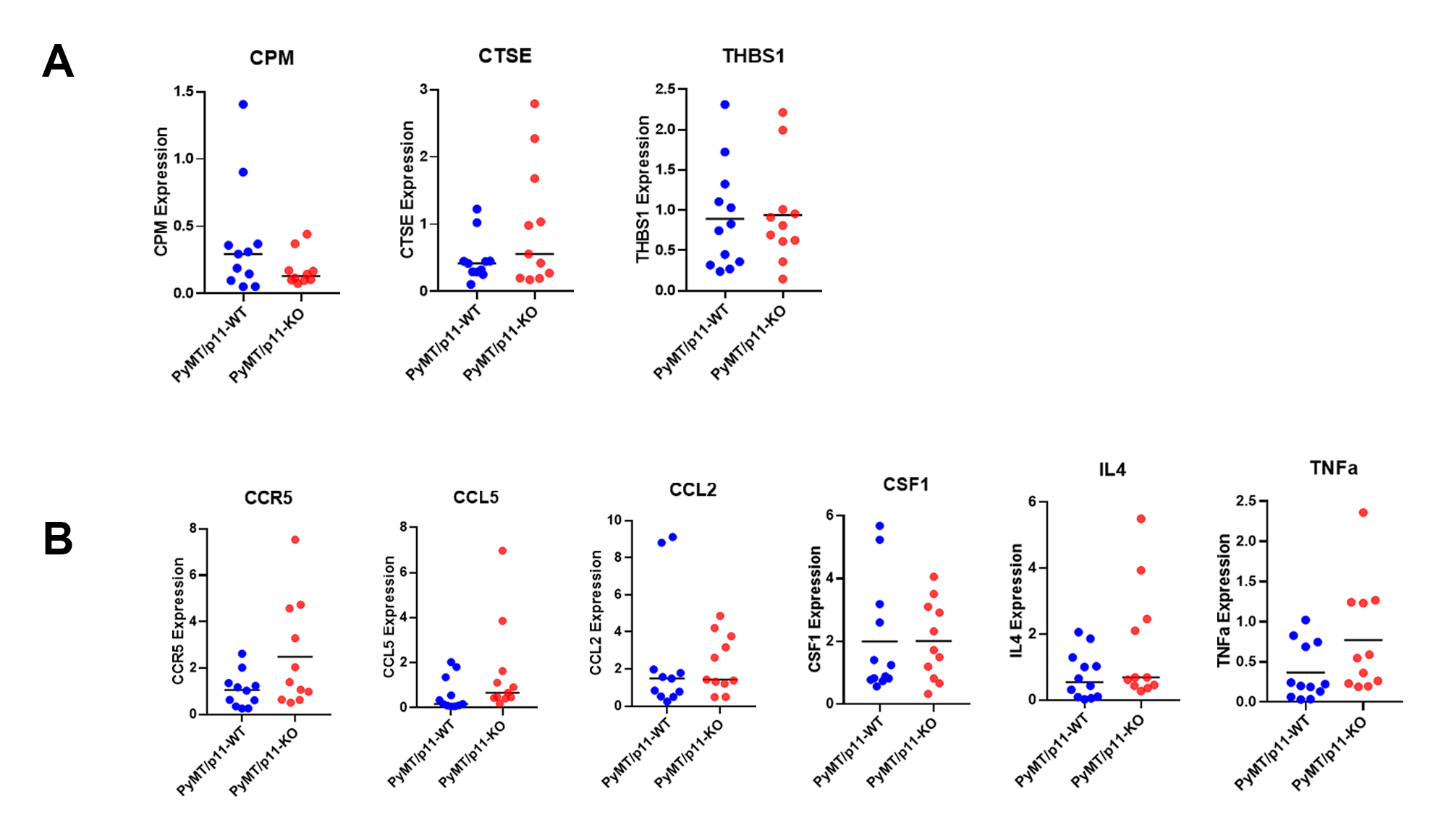


**Figure S6.** Gene expression profiling of PyMT/p11-WT and PyMT/p11-KO tumors. (A) Validation of the top 9 differentially expressed genes using qPCR (10-11 mice per group). Significance was determined by an unpaired t-test. Only the genes not significantly altered are shown. (B) Loss of p11 in PyMT tumors results in differential cytokine expression profile. We performed quantitative RT-PCR on tumors isolated from PyMT/p11-WT and PyMT/p11KO mice (10-11 mice per group), using a CFX96 or CFX384 Touch Real-Time PCR Detection System (BioRad, Mississauga, ON, Canada). Relative mRNA expression was log-2 transformed before plotting and statistical analysis. Significance was determined by an unpaired, t-test. Only those cytokines not significantly altered are shown here.


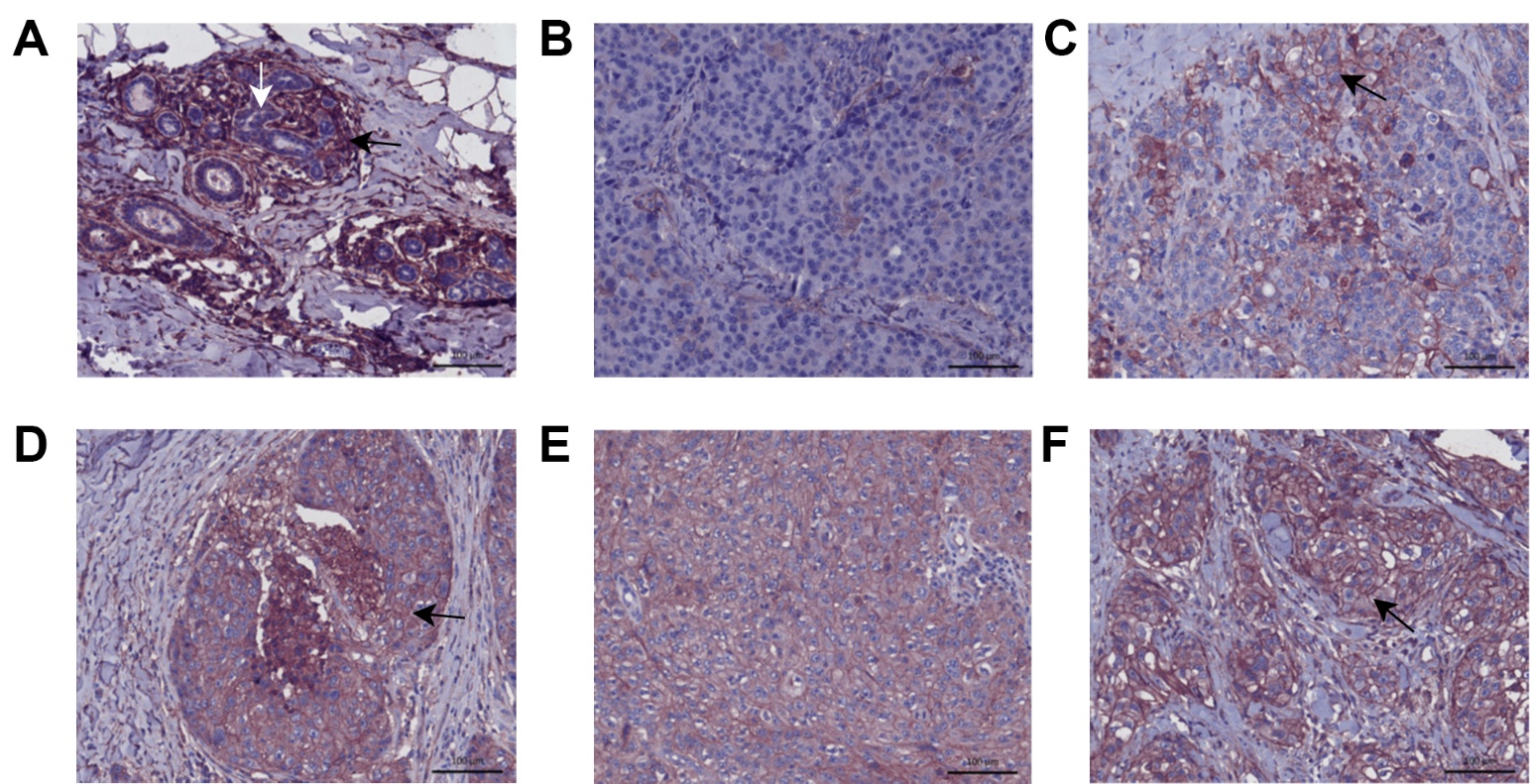


**Figure S7.** Immunohistochemical staining of p11 in human tissue sections. Normal breast tissues, low grade (LG), high grade (HG), ductal carcinoma in situ (DCIS), IDC/Invasive, ER+, HER2+, and Triple-negative (TN) tumors were obtained from Queen Elizabeth II Health Sciences Centre, Nova Scotia Health Authority (*see Materials and Methods*). The sections were stained with anti-p11 antibody (ProteinTech, Rosemont, IL, USA) as described in Materials and Methods). The representative image was captured using Zeiss Axio Imager Z1 W/ color camera at 20X magnification. Representative images from (A) normal – white arrow indicates the epithelial cells lining the duct, black arrow indicates stromal cells (B) tumors/cells staining negative for p11 (C) low H-score, black arrow indicates pockets of positive membrane staining (D) DCIS staining, high H-score, black arrow indicates the positive membrane staining € moderate staining in invasive carcinoma and (F)strong staining in invasive carcinoma, black arrow indicates the positive membrane staining. Scale bar – 100 µm**
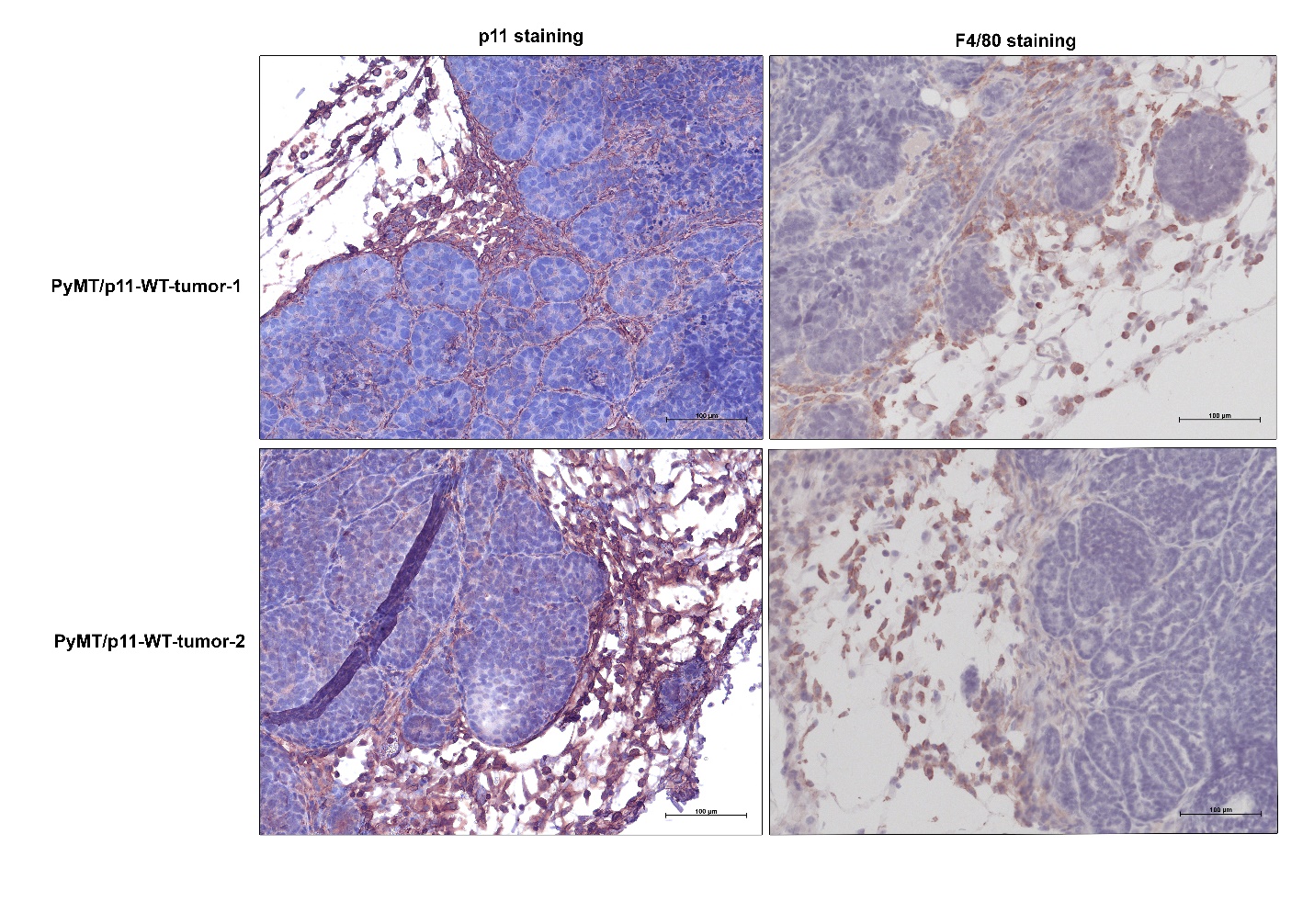
****Figure S8.** Immunohistochemical staining of p11 and F4/80 in the PyMT/p11-WT tumors. To determine if p11 expression is localized to the macrophage population of the stromal cells, we performed IHC staining for p11 and F4/80 as described in the Materials and Methods. Two representative (different) fields of p11 and F4/80 stained sections from the same PyMT/p11-WT tumor samples are shown. Scale bar – 100 µm.

**Table S1.** Sequences of primers employed in the q-PCR validation of microarray hits, q-PCR expression of cytokine in PyMT tumors and genotyping for PyMT and p11.

| ALDH1A2.F | TCCCTAAATGGCGGTAAGCC |
| --- | --- |
| ALDH1A2.R | AGCCCAGCCTGCATAATACC |
| AREG.F | TTGCTGCTGGTCTTAGGCTC |
| AREG.R | TGGTCCCCAGAAAGCGATTC |
| CDH19.F | GAAAGCTGAGAAGGCTCGGA |
| CDH19.R | AACAAATGGTCTCCACACCCA |
| CPM.F | GAGGCGTTCCTAAAGAGCGT |
| CPM.R | TGTTCCTTTGGAGTCTGCCC |
| CTSE.F | GCCACACCCATTGATGGAGA |
| CTSE.R | GTCCGGCAGGATGTAGTCAG |
| MUC1.F | AGTACCAAGCGTAGCCCCTA |
| MUC1.R | CACCACAGCTGGGTTGGTAT |
| THBS1.F | GGTACAGACAAGGACCTGCC |
| THBS1.R | TGATCACACCGTCACCACAG |
| TNC.F | GAGACCTCCTACCGCCAAAC |
| TNC.R | GGCCTCGGGTGTTGTTTTTC |
| S100A8.F | TGGAGAAGGCCTTGAGCAAC |
| S100A8.R | AGTCATTCTTGTAGAGGGCATGG |
| S100A10.Primer 1(genotyping) | CATTCAGAGGTGAACCCTGCTGAGGG |
| S100A10.Primer 2(genotyping) | CCTGTCAGCCACTCTATATGC |
| S100A10.Primer 3(genotyping) | GGCCAGCTCATTCCTCCCACTCATG |
| PyMT.F (genotyping) | GGAAGCAAGTACTTCACAAGGG |
| PyMT.R (genotyping) | GGAAAGTCACTAGGAGCAGGG |
| GAPDH.F | GCGAGACCCCACTAACATCA |
| GAPDH.R | GGCGGAGATGATGACCCTTT |
| RPL10.F | GCGACCTGGAAGTCCAACTA |
| RPL10.R | ACGTTGTCTGCTCCCACAAT |
| CCR5.F | CTGCTGCCTAAACCCTGTCA |
| CCR5.R | TGCAAAAGCGTTTGACCATGT |
| CSF2.F | CTCCGGAAACGGACTGTGAA |
| CSF2.R | TGCATTCAAAGGGGATATCAGTCA |
| IL6.F | TCCAGTTGCCTTCTTGGGAC |
| IL6.R | TGAAGTCTCCTCTCCGGACTT |
| TNFα.F | AGCCGATGGGTTGTACCTTG |
| TNFα.R | ATAGCAAATCGGCTGACGGT |
| mCCL2.F | CAGATGCAGTTAACGCCCCA |
| mCCL2.R | GCTTCTTTGGGACACCTGCT |
| IL4.F | CGTCTGTAGGGCTTCCAAGG |
| IL4.R | AGGCATCGAAAAGCCCGAA |
| IL12α.F | CCCTTGCCCTCCTAAACCAC |
| IL12α.R | GACTGGCTAAGACACCTGGC |
| mCCL5.F | TGCTCCAATCTTGCAGTCGT |
| mCCL5.R | TTGAACCCACTTCTTCTCTGGG |
| mCSF1.F | AGACCAGGAACAGCTGGATGA |
| mCSF1.R | CTCTCGGTGGCGTTAGCATT |
| PyMT.F (q-PCR) | CTCCAACAGATACACCCGCACATACT |
| PyMT.R (q-PCR) | GCTGGTCTTGGTCGCTTTCTGGATAC |
| IFNγ.F | CAGCACTCGAATGTGTCAGG |
| IFNγ.R | GAAGCACCAGGTGTCAAGTC |

The table is the list of forward and reverse primers used for validation of significant hits (9 genes) from the gene expression profiling of PyMT mouse tumors, primers for cytokine expression analysis in the PyMT tumors, and primers for genotyping mice for PyMT and p11 expression. .

**Table S2.** Antibodies used for immunoblotting and IHC.

| **Antibody** | **Company** | **Catalogue #** | **Dilution** | **Secondary** | **Antigen retrieval** | **Chromogen** |
| --- | --- | --- | --- | --- | --- | --- |
| P11 (Immunoblot) | R and D Systems | AF2377 | 1:2000 | Anti-goat secondary, IR conjugated | N/A | N/A |
| P11 (IHC) | Proteintech | 11250-1-AP | 1:200 (Mouse tissues)  1:400 (human tissues) | Anti-rabbit Envison (Dako) | Pressure cooker, Citrate buffer pH 6.0 | DAB |
| α-smooth muscle actin | Sigma (1A4) | A2547 | 1:10,000 | Mouse-on-Mouse HRP Polymer (Biocare) | Pressure cooker, EDTA de-cloacker (Biocare) | DAB |
| Ki67 | Abcam | Ab16667 | 1:50 | Anti-rabbit Envison (Dako) | Pressure cooker, Citrate buffer pH 6.0 | DAB |
| CD31 | Abcam | Ab28364 | 1:100 | Anti-rabbit Envison (Dako) | Pressure cooker, Tris-EDTA buffer, pH 9.0 | DAB |
| F4/80 (BM8) | Thermofisher Scientific | 14-4801-85 | 1:10 | Rat-mouse-HRP polymer (Biocare) | Pronase (Biocare) | DAB |
| CD3 | Abcam | ab16669 | 1:50 | Anti-rabbit Envison (Dako) | Pressure cooker, Citrate buffer pH 6.0 | DAB |
| Tubulin (Immunoblot) | Sigma | T7816 | 1:40,000 | Anti-mouse-IR conjugated | N/A | N/A |

The table lists the various antibodies, catalog #, antibody source, dilutions, secondary antibodies, antigen retrieval, and chromogen used for IHC and immunoblots.

**S1 File.** Microarray hits for PyMT mouse tumor gene expression profiling. This file in an excel spreadsheet listing all the hits obtained from gene expression profiling of PyMT tumors.

| 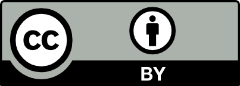 | © 2020 by the authors. Submitted for possible open access publication under the terms and conditions of the Creative Commons Attribution (CC BY) license (http://creativecommons.org/licenses/by/4.0/). |
| --- | --- |
